# Supplementary material for: Derivatives and inverse of cascaded linear+nonlinear neural models
Source: PLoS One. 2018 Oct 15;13(10):e0201326. doi: 10.1371/journal.pone.0201326 (PMC6188639; doi:10.1371/journal.pone.0201326)
Supplement: S1 File — (PDF) [file pone.0201326.s001.pdf]

Supporting Information file S1:
S1. A cascaded linear+nonlinear vision model

The illustrative model considered in the Discussion was originally intended to provide a psychophysically meaningful alternative to the *modular concept* in Structural Similarity measures (SSIM). The authors of SSIM suggest a separate consideration of luminance, contrast and structure [1], which is a sensible approach, but the definition of such factors has no obvious perceptual meaning in SSIM. The idea for a perceptual alternative to SSIM proposed in [2] was addressing one psychophysical factor at a time (i.e. also a modular approach), by using a cascade of linear-nonlinear transforms. In this paper we consider a cascade of 4 L+NL layers, each focused on a different psychophysical factor:

Layer $S^{(1)}$ linear spectral integration to compute luminance and opponent tristimulus channels, and nonlinear brightness/color response.

Layer $S^{(2)}$ definition of local contrast by using linear filters and divisive normalization.

Layer $S^{(3)}$ linear LGN-like contrast sensitivity filter and nonlinear local contrast masking in the spatial domain.

Layer $S^{(4)}$ linear V1-like wavelet decomposition and nonlinear divisive normalization to account for orientation and scale-dependent masking.

Here we extend previous results by considering two extra layers (1-st and 4-th layers were not considered in [2]). Following the suggestion in [3], here we use the canonical Divisive Normalization for each of these layers. Below we present the expressions of the *forward transforms*, their *derivatives w.r.t. the stimulus*, their *derivatives w.r.t. the parameters*, and their *inverses*.

S1.1 Forward transforms

The different layers are almost isomorphic: while the 3rd and 4th layers follow the divisive normalization expression introduced in the main text, the 1st and 2nd layers only differ in that they operate on positive signals (luminance and brightness respectively), and in a global normalization constant in the 1st layer.

Layer 1: Brightness from Radiance

$$\begin{aligned} \mathcal{L}^{(1)} \equiv \quad & \boldsymbol{y}^1 = L^1 \cdot \boldsymbol{x}^0 \\ \mathcal{N}^{(1)} \equiv \quad & \boldsymbol{x}^1 = K(\boldsymbol{y}^1) \cdot \mathbb{D}^{-1}_{\left(\boldsymbol{b}^1 + H^1 \cdot \boldsymbol{y}^{1\gamma^1}\right)} \cdot \boldsymbol{y}^{1\gamma^1} \end{aligned} \quad \text{(S1.1)}$$

where, L^1 is a matrix with the color matching functions for each spatial location. In particular, restricting ourselves to achromatic information, the only required color matching function would be the spectral sensitivity V_λ [4,5], leading to the luminance in each spatial location. The global scaling matrix $K(\boldsymbol{y}^1) = \kappa \left(\mathbb{D}_{\boldsymbol{b}^1} + \mathbb{D}_{\left(\frac{\beta}{d}\mathbb{1}\cdot\boldsymbol{y}^{1\gamma^1}\right)} + I \right)$, just ensures that the maximum brightness value (for normalized luminance equal to 1) is κ. The role of the interaction kernel in the denominator $H^1 = \left(\frac{\beta}{d}\mathbb{1} + I\right)$, where $\mathbb{1}$ is the all-ones $d \times d$ matrix, and I is the identity matrix, is setting the anchor for the brightness adaptation. With this kernel in the denominator the anchor luminance is

related to the average luminance energy $\left(\boldsymbol{b}^1 + \frac{\beta}{d}\mathbb{1}\cdot{\boldsymbol{y}^1}^{\gamma^1}\right)$. The effect of this nonlinear transform is a Weber-like adaptive saturation [6]. Similar nonlinear behavior can be assumed for the opponent chromatic channels [5, 7, 8], but we didnt implemented the color version of the model.

Layer 2: Contrast from Brightness

$$\begin{aligned} \mathcal{L}^{(2)} \equiv \qquad \boldsymbol{y}^2 &= L^2 \cdot \boldsymbol{x}^1 \\ \mathcal{N}^{(2)} \equiv \qquad \boldsymbol{x}^2 &= \mathbb{D}^{-1}_{(\boldsymbol{b}^2 + H^2\cdot\boldsymbol{y}^2)} \cdot \boldsymbol{y}^2 \end{aligned} \tag{S1.2}$$

where the linear stage computes the deviation of point-wise brightness with regard to the local brightness through $L^2 = I - \mathcal{H}^n$, and this kernel in the *numerator*, $\mathcal{H}^n$, represents the convolution by a two-dimensional Gaussian (as in Eq. 33 of the main text). The normalization through $H^2 = \mathcal{H}^d \cdot (I - \mathcal{H}^n)^{-1}$, where the kernel in the *denominator*, $\mathcal{H}^d$, is another two-dimensional Gaussian kernel, leads to the standard definition of contrast: normalization of the deviation of brightness by the local brightness.

Layer 3: Contrast sensitivity and spatial masking

$$\begin{aligned} \mathcal{L}^{(3)} \equiv \qquad \boldsymbol{y}^3 &= L^3 \cdot \boldsymbol{x}^2 \\ \mathcal{N}^{(3)} \equiv \qquad \boldsymbol{x}^3 &= \mathbb{D}_{\text{sign}(\boldsymbol{y}^3)} \cdot \mathbb{D}^{-1}_{\left(\boldsymbol{b}^3 + H^3\cdot|\boldsymbol{y}^3|^{\gamma^3}\right)} \cdot |\boldsymbol{y}^3|^{\gamma^3} \end{aligned} \tag{S1.3}$$

where L^3 is the convolution matrix equivalent to the application of a Contrast Sensitivity Function (CSF) [9]. The rows of this matrix consist of displaced versions of center-surround (LGN-like) receptive fields (impulse response of the CSF [10]). The kernel in the denominator, H^3, represents the convolution by another two-dimensional Gaussian that computes the local contrast energy that masks the responses in high-energy environments.

Layer 4: Wavelet analysis and frequency masking

$$\begin{aligned} \mathcal{L}^{(4)} \equiv \qquad \boldsymbol{y}^4 &= L^4 \cdot \boldsymbol{x}^3 \\ \mathcal{N}^{(4)} \equiv \qquad \boldsymbol{x}^4 &= \mathbb{D}_{\text{sign}(\boldsymbol{y}^4)} \cdot \mathbb{D}^{-1}_{\left(\boldsymbol{b}^4 + H^4\cdot|\boldsymbol{y}^4|^{\gamma^4}\right)} \cdot |\boldsymbol{y}^4|^{\gamma^4} \end{aligned} \tag{S1.4}$$

where L^4 is the matrix of Gabor-like receptive fields corresponding to V1-like sensors [11]. The kernel in the denominator, H^4, represents the masking interaction between sensors tuned to different space, frequency and orientation [12].

S1.2 Derivatives

Due to the slight differences in the transforms for the 1st and 2nd layers, the derivatives and inverses are slightly different from Results I, II and III presented in the main text. However, here we simply list the corresponding expressions because their derivation is straightforward using the methods described to get Results I, II and III. Since the formulation of the 3rd and 4th layers is the one given in the main text, the corresponding expressions will not be repeated here.

Derivatives w.r.t stimulus.

$$\nabla_{\boldsymbol{y}^1}\mathcal{N}^{(1)} = K(\boldsymbol{y}^1)\cdot\left[\mathbb{D}^{-1}_{\mathcal{D}^{(1)}(\boldsymbol{y}^1)} - \mathbb{D}_{\left(\frac{\boldsymbol{y}^{1\gamma^1}}{\mathcal{D}^{(1)}(\boldsymbol{y}^1)^2}\right)}\cdot H^1\right]\cdot\mathbb{D}_{\left(\gamma^1\boldsymbol{y}^{1\gamma^1-1}\right)} + \frac{\kappa\beta}{d}\mathbb{D}_{\left(\frac{\boldsymbol{y}^{1\gamma^1}}{\mathcal{D}^{(1)}(\boldsymbol{y}^1)}\right)}\cdot\mathbb{1}\cdot\mathbb{D}_{\left(\gamma^1\boldsymbol{y}^{1\gamma^1-1}\right)} \tag{S1.5}$$

$$\nabla_{\boldsymbol{y}^2}\mathcal{N}^{(2)} = \left[\mathbb{D}^{-1}_{\mathcal{D}^{(2)}(\boldsymbol{y}^2)} - \mathbb{D}_{\left(\frac{\boldsymbol{y}^{2}}{\mathcal{D}^{(2)}(\boldsymbol{y}^2)^2}\right)}\cdot H^2\right] \tag{S1.6}$$

Where, as in the main text, $\mathcal{D}^{(i)}$, stands for the denominator corresponding to the normalization in the i-th layer.

Derivatives w.r.t. the semisaturation $\boldsymbol{b}$

$$\nabla_{\boldsymbol{b}^1}\mathcal{N}^{(1)} = -K(\boldsymbol{y}^1)\cdot\mathbb{D}_{\boldsymbol{y}^{1\gamma^1}}\cdot\mathbb{D}^{-2}_{\mathcal{D}^{(1)}(\boldsymbol{y}^1)} + \kappa\mathbb{D}_{\boldsymbol{y}^{1\gamma^1}}\cdot\mathbb{D}^{-1}_{\mathcal{D}^{(1)}(\boldsymbol{y}^1)} \tag{S1.7}$$

$$\nabla_{\boldsymbol{b}^2}\mathcal{N}^{(2)} = -\mathbb{D}_{\boldsymbol{y}^2}\cdot\mathbb{D}^{-2}_{\mathcal{D}^{(2)}(\boldsymbol{y}^2)} \tag{S1.8}$$

Derivatives w.r.t. the excitation-inhibition exponent γ

$$\nabla_{\gamma^1}\mathcal{N}^{(1)} = \left[K(\boldsymbol{y}^1)\cdot\mathbb{D}^{-1}_{\mathcal{D}^{(1)}(\boldsymbol{y}^1)}\cdot\left[\mathbb{D}_{\log\boldsymbol{y}^1} - \mathbb{D}^{-1}_{\mathcal{D}^{(1)}(\boldsymbol{y}^1)}\cdot\mathbb{D}_{\left(H^1\cdot\mathbb{D}_{\left(\boldsymbol{y}^{1\gamma^1}\right)}\cdot\log\boldsymbol{y}^1\right)}\right] + \frac{\kappa\beta}{d}\mathbb{D}_{\left(\frac{\boldsymbol{y}^{1\gamma^1}}{\mathcal{D}^{(1)}(\boldsymbol{y}^1)}\right)}\cdot\mathbb{1}\cdot\mathbb{D}_{\log\boldsymbol{y}^1}\right]\cdot\boldsymbol{y}^{1\gamma^1} \tag{S1.9}$$

Derivatives w.r.t. the global scaling constant κ

$$\nabla_{\kappa}\mathcal{N}^{(1)} = \left(\mathbb{D}_{\boldsymbol{b}^1} + \mathbb{D}_{\left(\frac{\beta}{d}\mathbb{1}\cdot\boldsymbol{y}^{1\gamma^1}\right)} + I\right)\cdot\mathbb{D}^{-1}_{\left(\boldsymbol{b}^1 + H^1\cdot\boldsymbol{y}^{1\gamma^1}\right)}\cdot\boldsymbol{y}^{1\gamma^1} \tag{S1.10}$$

Derivatives w.r.t. the parameters of kernels L and H. The parameter of the normalization kernel of the 1st stage, H^1, is the weight, β, of the adaptation anchor for the luminance. The derivative w.r.t. β is:

$$\nabla_{\beta}\mathcal{N}^{(1)} = \frac{1}{d}\mathbb{D}^{-1}_{\mathcal{D}^{(1)}(\boldsymbol{y}^1)}\cdot\left[\kappa\mathbb{D}_{\left(\mathbb{1}\cdot\boldsymbol{y}^{1\gamma^1}\right)} - K(\boldsymbol{y}^1)\cdot\mathbb{D}_{\left(\frac{\boldsymbol{y}^{1\gamma^1}}{\mathcal{D}^{(1)}(\boldsymbol{y}^1)}\right)}\cdot\mathbb{1}\right]\cdot\boldsymbol{y}^{1\gamma^1} \tag{S1.11}$$

Contrast computation in the 2nd layer depends on the application of two Gaussian kernels: one in the linear stage, $L^2 = \mathbb{1} - \mathcal{H}^n$, and another in the nonlinear stage, $H^2 = \mathcal{H}^d\cdot(\mathbb{1} - \mathcal{H}^n)^{-1}$. The rows of each of these kernels depends on the corresponding width and amplitude.

For the set of widths and amplitudes of $\mathcal{H}^n$, $\boldsymbol{\sigma}^n$ and $\boldsymbol{c}^n$:

$$\nabla_{\boldsymbol{\sigma}^n}\mathcal{L}^{(2)} = -\nabla_{\boldsymbol{y}^2}\mathcal{N}^{(2)}\cdot\mathrm{diag}\left[\begin{pmatrix}\boldsymbol{x}^{1\top}\\ \boldsymbol{x}^{1\top}\\ \vdots\\ \boldsymbol{x}^{1\top}\end{pmatrix}\cdot F^n\right] \tag{S1.12}$$

where, $F^n_{kk'} = c^n_k\,\frac{dp_{k1}dp_{k2}}{2\pi\ \sigma^{n\,5}_k}\ \left(\Delta^2_{kk'} - 2\,\sigma^{n\,2}_k\right)\ e^{-\frac{\Delta^2_{kk'}}{2\ \sigma^{n\,2}_k}}$. In this expression, $\Delta^2_{kk'}$ and $dp_{k1}dp_{k2}$ have the same meaning used in the main text (Eq. 33) in the context of the Gaussian kernels.

$$\nabla_{\boldsymbol{c}^n}\mathcal{L}^{(2)} = -\nabla_{\boldsymbol{y}^2}\mathcal{N}^{(2)} \cdot \mathrm{diag}\left[\begin{pmatrix} \boldsymbol{x}^{1\top} \\ \boldsymbol{x}^{1\top} \\ \vdots \\ \boldsymbol{x}^{1\top} \end{pmatrix} \cdot G^n\right] \tag{S1.13}$$

where, $G^n_{kk'} = \frac{dp_{k1}dp_{k2}}{2\pi\ \sigma^{n\,2}_k}\ e^{-\frac{\Delta^2_{kk'}}{2\ \sigma^{n\,2}_k}}$.

Similarly, for the set of widths and amplitudes of $\mathcal{H}^d$, $\boldsymbol{\sigma}^d$ and $\boldsymbol{c}^d$:

$$\nabla_{\boldsymbol{\sigma}^d}\mathcal{N}^{(2)} = -\mathrm{diag}\left[\mathbb{D}_{\boldsymbol{y}^2} \cdot \mathbb{D}^{-2}_{\mathcal{D}^{(2)}(\boldsymbol{y}^2)} \cdot \begin{pmatrix} \left((\mathbb{1}-\mathcal{H}^n)^{-1}\cdot\boldsymbol{y}^2\right)^\top \\ \left((\mathbb{1}-\mathcal{H}^n)^{-1}\cdot\boldsymbol{y}^2\right)^\top \\ \vdots \\ \left((\mathbb{1}-\mathcal{H}^n)^{-1}\cdot\boldsymbol{y}^2\right)^\top \end{pmatrix} \cdot F^d\right] \tag{S1.14}$$

where, $F^d_{kk'} = c^d_k\ \frac{dp_{k1}dp_{k2}}{2\pi\ \sigma^{d\,5}_k}\ \left(\Delta^2_{kk'} - 2\,\sigma^{d\,2}_k\right)\ e^{-\frac{\Delta^2_{kk'}}{2\ \sigma^{d\,2}_k}}$.

$$\nabla_{\boldsymbol{c}^d}\mathcal{N}^{(2)} = -\mathrm{diag}\left[\mathbb{D}_{\boldsymbol{y}^2} \cdot \mathbb{D}^{-2}_{\mathcal{D}^{(2)}(\boldsymbol{y}^2)} \cdot \begin{pmatrix} \left((\mathbb{1}-\mathcal{H}^n)^{-1}\cdot\boldsymbol{y}^2\right)^\top \\ \left((\mathbb{1}-\mathcal{H}^n)^{-1}\cdot\boldsymbol{y}^2\right)^\top \\ \vdots \\ \left((\mathbb{1}-\mathcal{H}^n)^{-1}\cdot\boldsymbol{y}^2\right)^\top \end{pmatrix} \cdot G^d\right] \tag{S1.15}$$

where, $G^d_{kk'} = \frac{dp_{k1}dp_{k2}}{2\pi\ \sigma^{d\,2}_k}\ e^{-\frac{\Delta^2_{kk'}}{2\ \sigma^{d\,2}_k}}$.

S1.3 Inverses

The inverse of the 2nd layer is simpler than Result III,

$$\boldsymbol{y}^2 = \left(\mathbb{1} - \mathbb{D}_{\boldsymbol{x}^2}\cdot H^2\right)^{-1}\cdot\mathbb{D}_{\boldsymbol{x}^2}\cdot\boldsymbol{b}^2 \tag{S1.16}$$

On the contrary, the inverse of the 1st layer may require an iterative process. As stated above, the saturation in Eq. S1.1 depends on an anchor for the luminance adaptation. In the forward transform this anchor is computed through the average of the known luminance in the considered image. But of course, the luminance values are not known when computing the inverse. If there is no other way to assume certain adaptation state (or average luminance), the solution may be achieved iteratively. First, assume certain reasonable average luminance to estimate the scaling matrix K. Then, use this estimated K to compute a first estimation of the luminance from known the brightness response and the analytic inverse. This new estimation of the luminance vector can be used again for a better estimation of K, and this process can be iterated. By using the *energy* notation used in the main text, $\boldsymbol{e}^1 = \boldsymbol{y}^{1\,\gamma^1}$, and using n as iteration index,

$$\begin{aligned} \boldsymbol{e}^1_n \quad &\rightarrow \quad K_n(\boldsymbol{e}^1_n) = \kappa\left(\mathbb{D}_{\boldsymbol{b}^1} + \mathbb{D}_{\left(\frac{\beta}{d}\mathbb{1}\cdot\boldsymbol{e}^1_n\right)} + I\right) \\ \boldsymbol{e}^1_{n+1} \quad &= \quad \left(\mathbb{1} - \mathbb{D}_{\left(K_n^{-1}\cdot\boldsymbol{x}^1\right)}\cdot H^1\right)^{-1}\cdot\mathbb{D}_{\left(K_n^{-1}\cdot\boldsymbol{x}^1\right)}\cdot\boldsymbol{b}^1 \end{aligned} \tag{S1.17}$$

Note: alternative formulations in certain layers. The illustrative model used in the Discussion, described in this supporting information, and implemented in the toolbox associated to the paper, is based on a cascade of almost isomorphic L+NL transforms (see Eqs. S1.1 - S1.4). However, as stated in the main text, this implementation based on isomorphic divisive normalization transforms is not the only possible choice.

The main text cites specific alternatives for the brightness transform (the two-gamma curve [13–15]) and for the interaction between V1-like sensors (the Wilson-Cowan model [16, 17]). The alternative transforms and their jacobians w.r.t. the stimulus were presented in the main text in sections 2.1 and 2.2 The analytic inverse of the Wilson-Cowan interaction was presented in section 2.4.

The inverse of the two-gamma nonlinearity was not addressed there because, given the coupling between the input and the exponent (see Eq. 14),Eq. 13 has no analytical inverse. Nevertheless, iterative approximations to the actual luminance value can be obtained in the following way. First one makes an initial guess of the exponent (for instance the average value between the two extremes), and then one obtains the first guess for the luminance assuming this approximate exponent. Afterwards, the estimate of the exponent is recomputed from the new luminance estimate, and so on. At the n-th iteration,

$$\begin{aligned} n &= 0 \begin{cases} \gamma^1_0 = \frac{1}{2}(\gamma_L + \gamma_H) \\ \boldsymbol{y}^1_0 = \boldsymbol{x}^{1\,\frac{1}{\gamma^1_0}} \end{cases} \\ n &> 0 \begin{cases} \gamma^1_n = \gamma^1(\boldsymbol{y}^1_{(n-1)}) \\ \boldsymbol{y}^1_n = \boldsymbol{x}^{1\,\frac{1}{\gamma^1_n}} \end{cases} \end{aligned} \quad \text{(S1.18)}$$

where thee subindex n indicates the iteration and $\gamma^1(\boldsymbol{y}^1)$ is computed using Eq. 14. Note that, given the singularity at the origin of the jacobian of the two-gamma response (see the discussion made after Eq. 27 in the main text), the above only holds for big enough luminance, $\boldsymbol{y}^1 \in [\epsilon^{\gamma^1(\epsilon)}, 1]$. For small luminance values, $\boldsymbol{y}^1 \in [0, \epsilon^{\gamma^1(\epsilon)}]$, it holds the robust regime introduced to solve the mentioned singularity, and this parabolic expression is invertible:

$$\boldsymbol{y}^1 = \frac{1}{\sqrt{a_1}}\sqrt{\boldsymbol{x}^1 + \frac{a_2^2}{4a_1}} - \frac{a_2}{2a_1} \quad \text{(S1.19)}$$

References

1. Wang Z, Bovik AC, Sheikh HR, Simoncelli EP. Image quality assessment: from error visibility to structural similarity. IEEE Trans Im Proc. 2004;13(4):600–612.

2. Malo J, Simoncelli E. Geometrical and statistical properties of vision models obtained via maximum differentiation. In: SPIE Electronic Imaging. International Society for Optics and Photonics; 2015. p. 93940L–93940L.

3. Carandini M, Heeger DJ. Normalization as a canonical neural computation. Nature Rev Neurosci. 2012;13(1):51–62.

4. Wyszecki G, Stiles WS. Color Science: Concepts and Methods, Quantitative Data and Formulae. New York: John Wiley & Sons; 1982.

5. Fairchild MD. Color appearance models. Wiley; 2013.

6. Abrams AB, Hillis JM, Brainard DH. The Relation Between Color Discrimination and Color Constancy: When Is Optimal Adaptation Task Dependent? Neural Computation. 2007;19(10):2610–2637.

7. Stockman A, Brainard DH. Color vision mechanisms. In: Bass M, editor. OSA Handbook of Optics (3rd. Ed.). NY: McGraw-Hill; 2010. p. 147–152.

8. Laparra V, Jiménez S, Camps-Valls G, Malo J. Nonlinearities and adaptation of color vision from sequential principal curves analysis. Neural Comp. 2012;24(10):2751–2788.

9. Campbell FW, Robson JG. Application of Fourier Analysis to the Visibility of Gratings. Journal of Physiology. 1968;197:551–566.

10. Martinez-Uriegas E. Color detection and color contrast discrimination thresholds. Proc OSA Meeting. 1997; p. 81.

11. Simoncelli EP, Adelson EH. Subband Transforms. In: Woods JW, editor. Subband image coding. Norwell, MA: Kluwer Academic Publishers; 1990. p. 143–192.

12. Watson AB, Solomon JA. A model of visual contrast gain control and pattern masking. JOSA A. 1997;14:2379–2391.

13. Cyriac P, Bertalmio M, Kane D, Vazquez-Corral J. A tone mapping operator based on neural and psychophysical models of visual perception. Proc IS&T/SPIE Electronic Imaging. 2015;9394. doi:10.1117/12.2081212.

14. Cyriac P, Kane D, Bertalmio M. Optimized Tone Curve for In-Camera Image Processing. IST Electronic Imaging Conference. 2016;13:1–7.

15. Kane D, Bertalmio M. System gamma as a function of image-and monitor-dynamic range. Journal of vision. 2016;16(6):4–4.

16. Wilson HR, Cowan JD. Excitatory and inhibitory interactions in localized populations of model neurons. Biophys J. 1972;12:1–24.

17. Cowan JD, Neuman J, van Drongelen W. Wilson–Cowan Equations for Neocortical Dynamics. J Math Neurosci. 2016;6(1):1–24.
