# Supplementary material for: Derivatives and inverse of cascaded linear+nonlinear neural models
Source: PLoS One. 2018 Oct 15;13(10):e0201326. doi: 10.1371/journal.pone.0201326 (PMC6188639; doi:10.1371/journal.pone.0201326)
Supplement: S2 File — (PDF) [file pone.0201326.s002.pdf]

Supporting Information file S2:

S2. Derivative of a linear function with regard to its parameters

The technical result in this section, namely Eq. S2.4, is necessary when deriving different analytical results of the paper: the Jacobian with regard to the parameters requires this sort of derivative in Eq. 21 of the main text (dependence with receptive fields) and in Eq. 32 of the main text (dependence with the kernel of the divisive normalization).

Given a general linear function depending on the rectangular matrix $L^i \in \mathbb{R}^{d_i \times d_{i-1}}$,

$$\boldsymbol{y}^i = \mathcal{L}^{(i)}(\boldsymbol{x}^{i-1}, L^i) = L^i \cdot \boldsymbol{x}^{i-1}$$

here we are interested in a matrix expression for its derivatives with regard to the parameters, L^i; i.e. we address the issue of the matrix form of,

$$\nabla_{L^i} \mathcal{L}^{(i)} = \frac{\partial \boldsymbol{y}^i}{\partial L^i}$$

In order to do so, go back to the individual dimensions for a moment: remember that we have d_i 1-dimensional functions like this,

$$y^i_k = \sum_{k'=1}^{d_{i-1}} L^i_{kk'} \; x^{i-1}_{k'}$$

where $k = 1, \ldots, d_i$. Therefore, $\nabla_{L^i} \boldsymbol{y}^i \in \mathbb{R}^{d_i \times (d_i \times d_{i-1})}$ because $\boldsymbol{y}^i \in \mathbb{R}^{d_i \times 1}$ and we have $d_i \times d_{i-1}$ elements in L^i. In this setting,

$$\frac{\partial y^i_k}{\partial L^i_{lm}} = \delta_{kl} \; x^{i-1}_m \tag{S2.1}$$

With a convenient rearrangement of L^i, it is possible to give a single matrix expression that summarizes the multiple element-wise derivatives in Eq. S2.1.

In particular, here we rearrange the parameters of the linear transform in the column vector $\boldsymbol{l}^i = \mathrm{vect}({L^i}^\top) \in \mathbb{R}^{(d_i \times d_{i-1}) \times 1}$. This arrangement is sensible in our context (feed-forward neural model) because of the following considerations. As stated above, the k-th row of the matrix, $L^i_{k\star}$, represents the linear weights of the k-th linear sensor in the i-th linear stage $\mathcal{L}^i$. In other words, $L^i_{k\star}$ represents the weighted connectivity or interaction of the k-th linear sensor with the previous stage. Given the scalar-product description of receptive fields [1, 2], one may say that each $L^i_{k\star}$ plays the role of a *receptive field* in the domain $\boldsymbol{x}^{i-1}$ because the k-th response is computed through these scalar products: $y^i_k = L^i_{k\star} \cdot \boldsymbol{x}^{i-1} = {\boldsymbol{x}^{i-1}}^\top \cdot {L^i_{k\star}}^\top$. With the proposed rearrangement, the column vector $\boldsymbol{l}^i$ is built by concatenating the transposed *receptive fields* of every linear sensor in the i-th layer,

$$\boldsymbol{l}^i = \mathrm{vect}({L^i}^\top) = \begin{pmatrix} \begin{pmatrix} L^i_{11} \\ L^i_{12} \\ \vdots \\ L^i_{1d_{i-1}} \end{pmatrix} \\ \begin{pmatrix} L^i_{21} \\ L^i_{22} \\ \vdots \\ L^i_{2d_{i-1}} \end{pmatrix} \\ \vdots \\ \begin{pmatrix} L^i_{d_i1} \\ L^i_{d_i2} \\ \vdots \\ L^i_{d_id_{i-1}} \end{pmatrix} \end{pmatrix} = \begin{pmatrix} \begin{pmatrix} \vdots \\ {L^i_{1\star}}^\top \\ \vdots \end{pmatrix} \\ \begin{pmatrix} \vdots \\ {L^i_{2\star}}^\top \\ \vdots \end{pmatrix} \\ \vdots \\ \begin{pmatrix} \vdots \\ {L^i_{d_i\star}}^\top \\ \vdots \end{pmatrix} \end{pmatrix} \tag{S2.2}$$

As a result, the small variations of the linear responses due to perturbations in the parameters of the linear transform (which are $\Delta y^i_k = {\boldsymbol{x}^{i-1}}^\top \cdot \Delta {L^i_{k\star}}^\top$), can be put in matrix form using the rearrangement $\boldsymbol{l}^i = \mathrm{vect}({L^i}^\top)$:

$$\begin{pmatrix} \Delta y^i_1 \\ \Delta y^i_2 \\ \vdots \\ \Delta y^i_{d_i} \end{pmatrix} = \begin{pmatrix} \left(\cdots {\boldsymbol{x}^{i-1}}^\top \cdots\right) & (\cdots \ 0 \ \cdots) & \cdots & (\cdots \ 0 \ \cdots) \\ (\cdots \ 0 \ \cdots) & \left(\cdots {\boldsymbol{x}^{i-1}}^\top \cdots\right) & \cdots & (\cdots \ 0 \ \cdots) \\ \vdots & \vdots & \ddots & \vdots \\ (\cdots \ 0 \ \cdots) & (\cdots \ 0 \ \cdots) & \cdots & \left(\cdots {\boldsymbol{x}^{i-1}}^\top \cdots\right) \end{pmatrix} \cdot \Delta \boldsymbol{l}^i \tag{S2.3}$$

since this leads to the appropriate scalar products. Identifying terms with the linear approximation in terms of the Jacobian,

$$\Delta \boldsymbol{y}^i = \nabla_{L^i} \mathcal{L}^{(i)} \cdot \Delta \boldsymbol{l}^i = \frac{\partial \boldsymbol{y}^i}{\partial L^i} \cdot \Delta \boldsymbol{l}^i$$

we see that, assuming the referred rearrangement of the parameters, the Jacobian we are interested in is given by the block diagonal matrix shown above.

In summary, given the linear transform $\boldsymbol{y}^i = L^i \cdot \boldsymbol{x}^{i-1}$, the Jacobian with regard to its parameters is:

$$\nabla_{L^i} \mathcal{L}^{(i)} = \frac{\partial \boldsymbol{y}^i}{\partial L^i} = \mathbb{B}^{d_i}_{({\boldsymbol{x}^{i-1}}^\top)} \tag{S2.4}$$

where $\mathbb{B}^m_{(A)}$ is a *block-diagonal* matrix built by replicating m times the matrix (or vector) A along the diagonal.

References

1. Olshausen B, Field D. Emergence of simple-cell receptive field properties by learning a sparse code for natural images. Nature. 1996;281:607–609. doi:10.1038/381607a0.
2. Ringach DL, Hawken MJ, Shapley R. Receptive field structure of neurons in monkey primary visual cortex revealed by stimulation with natural image sequences. Journal of Vision. 2002;2(1):2. doi:10.1167/2.1.2.
