# Supplementary material for: Derivatives and inverse of cascaded linear+nonlinear neural models
Source: PLoS One. 2018 Oct 15;13(10):e0201326. doi: 10.1371/journal.pone.0201326 (PMC6188639; doi:10.1371/journal.pone.0201326)
Supplement: S3 File — (PDF) [file pone.0201326.s003.pdf]

Supporting Information file S3:

S3. Derivation of the Jacobian with regard to the stimulus

Here we provide the proofs for two equations of the main text: (a) Eq. 24 for the canonical divisive normalization, and (b) Eq. 27 for the particular two-gamma nonlinearity. Remember the Jacobian of the particular Wilson-Cowan nonlinearity was already derived in the Results section (Eqs. 25 and 26).

Divisive normalization: proof of Eq. 24. Explicitly considering the sign and amplitude terms of the nonlinearity in Eq. 10, we can write, $\mathcal{N}^{(i)}(\boldsymbol{y}^i) = \mathbb{D}_{\mathrm{sign}(\boldsymbol{y}^i)} \cdot N^{(i)}(\boldsymbol{e}^i)$. Then, using the chain rule, we have:

$$\nabla_{\boldsymbol{y}^i}\mathcal{N}^{(i)} = \nabla_{\boldsymbol{y}^i}\,\mathrm{sign}(\boldsymbol{y}^i)\cdot\mathbb{D}_{N^{(i)}(\boldsymbol{e}^i)} + \mathbb{D}_{\mathrm{sign}(\boldsymbol{y}^i)}\cdot\nabla_{\boldsymbol{y}^i}N^{(i)}(\boldsymbol{e}^i)$$

where,

$$\nabla_{\boldsymbol{y}^i}\,\mathrm{sign}(\boldsymbol{y}^i)\cdot\mathbb{D}_{N^{(i)}(\boldsymbol{e}^i)} = 0,\ \text{ since } \mathrm{sign}(y^i_k) \text{ is constant } \forall y^i_k \neq 0, \text{ and } N^{(i)}(0) = 0$$

$$\nabla_{\boldsymbol{y}^i}N^{(i)}(\boldsymbol{e}^i) = \nabla_{\boldsymbol{e}^i}N^{(i)}(\boldsymbol{e}^i)\cdot\nabla_{\boldsymbol{y}^i}\boldsymbol{e}^i$$

where,

$$\nabla_{\boldsymbol{e}^i}N^{(i)}(\boldsymbol{e}^i) = \nabla_{\boldsymbol{e}^i}\boldsymbol{e}^i\ \cdot\mathbb{D}_{\left(\frac{1}{\mathcal{D}^{(i)}(\boldsymbol{e}^i)}\right)} + \mathbb{D}_{\boldsymbol{e}^i}\cdot\nabla_{\boldsymbol{e}^i}\frac{1}{\mathcal{D}^{(i)}(\boldsymbol{e}^i)}$$

where,

$$\nabla_{\boldsymbol{e}^i}\boldsymbol{e}^i = \mathbb{1}$$

$$\nabla_{\boldsymbol{e}^i}\frac{1}{\mathcal{D}^{(i)}(\boldsymbol{e}^i)} = -\mathbb{D}_{\frac{1}{\mathcal{D}^{(i)}(\boldsymbol{e}^i)^2}}\cdot\nabla_{\boldsymbol{e}^i}\mathcal{D}^{(i)}(\boldsymbol{e}^i) = -\mathbb{D}_{\frac{1}{\mathcal{D}^{(i)}(\boldsymbol{e}^i)^2}}\cdot H^i$$

therefore,

$$= \mathbb{D}_{\left(\frac{1}{\mathcal{D}^{(i)}(\boldsymbol{e}^i)}\right)} - \mathbb{D}_{\boldsymbol{e}^i}\cdot\mathbb{D}_{\frac{1}{\mathcal{D}^{(i)}(\boldsymbol{e}^i)^2}}\cdot H^i$$

$$\nabla_{\boldsymbol{y}^i}\boldsymbol{e}^i = \nabla_{|\boldsymbol{y}^i|}\boldsymbol{e}^i\cdot\nabla_{\boldsymbol{y}^i}|\boldsymbol{y}^i|$$

where,

$$\nabla_{|\boldsymbol{y}^i|}\boldsymbol{e}^i = \mathbb{D}_{\gamma^i|\boldsymbol{y}^i|^{\gamma^i-1}}$$

$$\nabla_{\boldsymbol{y}^i}|\boldsymbol{y}^i| = \mathbb{D}_{\mathrm{sign}(\boldsymbol{y}^i)},\ \text{ since the slope of } |\boldsymbol{y}^i| \text{ is } \mathrm{sign}(\boldsymbol{y}^i)$$

therefore,

$$= \mathbb{D}_{\gamma^i|\boldsymbol{y}^i|^{\gamma^i-1}}\cdot\mathbb{D}_{\mathrm{sign}(\boldsymbol{y}^i)}$$

as a result,

$$= \left[\mathbb{D}_{\left(\frac{1}{\mathcal{D}^{(i)}(\boldsymbol{e}^i)}\right)} - \mathbb{D}_{\frac{\boldsymbol{e}^i}{\mathcal{D}^{(i)}(\boldsymbol{e}^i)^2}}\cdot H^i\right]\cdot\mathbb{D}_{\gamma^i|\boldsymbol{y}^i|^{\gamma^i-1}}\cdot\mathbb{D}_{\mathrm{sign}(\boldsymbol{y}^i)}$$

finally, putting all the pieces together, we have Eq. 24.

Two-gamma nonlinearity: proof of Eq. 27. Given the separation in sign/amplitude, $\boldsymbol{x} = \mathbb{D}_{\mathrm{sign}(\boldsymbol{y})}\cdot|\boldsymbol{y}|^{\gamma(|\boldsymbol{y}|)}$, the derivative is,

$$\frac{\partial\boldsymbol{x}}{\partial\boldsymbol{y}} = \frac{\partial\boldsymbol{x}}{\partial|\boldsymbol{y}|}\cdot\frac{\partial|\boldsymbol{y}|}{\partial\boldsymbol{y}} = \left[\frac{\partial\,\mathrm{sign}(\boldsymbol{y})}{\partial|\boldsymbol{y}|}\cdot\mathbb{D}_{|\boldsymbol{y}|^{\gamma(|\boldsymbol{y}|)}} + \mathbb{D}_{\mathrm{sign}(\boldsymbol{y})}\cdot\frac{\partial\,|\boldsymbol{y}|^{\gamma(|\boldsymbol{y}|)}}{\partial|\boldsymbol{y}|}\right]\cdot\frac{\partial|\boldsymbol{y}|}{\partial\boldsymbol{y}}$$

where the first term in the parenthesis cancels for the same reasons stated in the previous proof, and the slope the magnitude is the sign, therefore,

$$\frac{\partial \boldsymbol{x}}{\partial \boldsymbol{y}} = \mathbb{D}_{\text{sign}(\boldsymbol{y})} \cdot \frac{\partial\, |\boldsymbol{y}|^{\gamma(|\boldsymbol{y}|)}}{\partial |\boldsymbol{y}|} \cdot \mathbb{D}_{\text{sign}(\boldsymbol{y})} = \frac{\partial\, |\boldsymbol{y}|^{\gamma(|\boldsymbol{y}|)}}{\partial |\boldsymbol{y}|}$$

where signs cancel out because all the matrices are diagonal and hence the product is commutative. Now, by calling $f = |\boldsymbol{y}|^{\gamma(|\boldsymbol{y}|)}$, our problem is computing $\frac{\partial f}{\partial |\boldsymbol{y}|}$. This notation is convenient since, taking the element-wise logarithm,

$$\log f = \gamma(|\boldsymbol{y}|) \odot \log |\boldsymbol{y}| \tag{S3.1}$$

and hence, applying the chain rule in the derivative of $\log f$ we have,

$$\frac{\partial \log f}{\partial |\boldsymbol{y}|} = \frac{\partial \log f}{\partial f} \cdot \frac{\partial f}{\partial |\boldsymbol{y}|} = \mathbb{D}_f^{-1} \cdot \frac{\partial f}{\partial |\boldsymbol{y}|} \quad \Rightarrow \quad \frac{\partial f}{\partial |\boldsymbol{y}|} = \mathbb{D}_f \cdot \frac{\partial \log f}{\partial |\boldsymbol{y}|} \tag{S3.2}$$

because the derivative matrices are diagonal, and hence all the products and quotients are Hadamard.

On the other hand, direct derivation of Eq. S3.1, leads to,

$$\frac{\partial \log f}{\partial |\boldsymbol{y}|} = \frac{\partial \gamma(|\boldsymbol{y}|)}{\partial |\boldsymbol{y}|} \cdot \mathbb{D}_{\log |\boldsymbol{y}|} + \mathbb{D}_{\gamma(|\boldsymbol{y}|)} \cdot \frac{\partial \log |\boldsymbol{y}|}{\partial |\boldsymbol{y}|} \tag{S3.3}$$

where, given the expression of the exponent γ, Eq. 14, its straightforward derivative:

$$\frac{\partial \gamma(|\boldsymbol{y}|)}{\partial |\boldsymbol{y}|} = \mathbb{D}_{\left((\gamma_H - \gamma_L) \cdot \frac{m\, |\boldsymbol{y}|^{(m-1)} \cdot \mu_1^m}{(\mu_1^m + |\boldsymbol{y}|^m)^2} \right)}$$

Then, plugging this derivative into Eq. S3.3, and the result into Eq. S3.2, we have Eq. 27 of the main text.
