# Supplementary material for: Derivatives and inverse of cascaded linear+nonlinear neural models
Source: PLoS One. 2018 Oct 15;13(10):e0201326. doi: 10.1371/journal.pone.0201326 (PMC6188639; doi:10.1371/journal.pone.0201326)
Supplement: S4 File — (PDF) [file pone.0201326.s004.pdf]

Supporting Information file S4:

S4. Derivation of the Jacobian with regard to the parameters

Here we provide the proofs for the Jacobian of the canonical divisive normalization wrt the parameters, namely for the Eqs. 30 - 35 in the main text.

Dependence on γ^i: Proof of Eq. 30. The divisive normalization, Eq. 10 in the main text, depends on the exponent γ^i through the vector of energies $\boldsymbol{e}^i = |\boldsymbol{y}^i|^{\gamma^i}$. Therefore,

$$\nabla_{\gamma^i}\mathcal{N}^{(i)} = -\mathbb{D}_{\mathrm{sign}(\boldsymbol{y}^i)} \cdot \mathbb{D}^{-2}_{\mathcal{D}^{(i)}(\boldsymbol{e}^i)} \cdot \frac{\partial \mathbb{D}_{\mathcal{D}^{(i)}(\boldsymbol{e}^i)}}{\partial \gamma^i} \cdot \boldsymbol{e}^i + \mathbb{D}_{\mathrm{sign}(\boldsymbol{y}^i)} \cdot \mathbb{D}^{-1}_{\mathcal{D}^{(i)}(\boldsymbol{e}^i)} \cdot \frac{\partial \boldsymbol{e}^i}{\partial \gamma^i} \tag{S4.1}$$

where, if we perturb γ, the vector in the diagonal gets perturbed, so,

$$\frac{\partial \mathbb{D}_{\mathcal{D}^{(i)}(\boldsymbol{e}^i)}}{\partial \gamma^i} = \mathbb{D}_{\frac{\partial \mathcal{D}^{(i)}(\boldsymbol{e}^i)}{\partial \gamma^i}} = \mathbb{D}_{\frac{\partial \mathcal{D}^{(i)}(\boldsymbol{e}^i)}{\partial \boldsymbol{e}^i} \cdot \frac{\partial \boldsymbol{e}^i}{\partial \gamma^i}} \tag{S4.2}$$

where the chain rule was also applied. Now, lets address the two derivatives in Eq. S4.2. First, given that $\mathcal{D}^{(i)}(\boldsymbol{e}^i) = \boldsymbol{b}^i + H^i \cdot \boldsymbol{e}^i$, the first term is,

$$\frac{\partial \mathcal{D}^{(i)}(\boldsymbol{e}^i)}{\partial \boldsymbol{e}^i} = H^i \tag{S4.3}$$

Regarding the second term in Eq. S4.2, $\nabla_{\gamma^i}\boldsymbol{e}^i$, by taking element-wise logarithms,

$$\log \boldsymbol{e}^i = \gamma^i \log |\boldsymbol{y}^i| \tag{S4.4}$$

this is convenient because, on the one hand, following a reasoning similar to the one used in Eq. S3.2, namely chain rule and direct derivation, we have:

$$\frac{\partial \boldsymbol{e}^i}{\partial \gamma^i} = \mathbb{D}_{\boldsymbol{e}^i} \cdot \frac{\partial \log \boldsymbol{e}^i}{\partial \gamma^i} = \mathbb{D}_{\boldsymbol{e}^i} \cdot \log |\boldsymbol{y}^i| \tag{S4.5}$$

Now, plugging Eqs. S4.3 and S4.5 into Eq. S4.2 and this in Eq. S4.1, we get Eq. 30. Note that $\nabla_{\gamma^i}\mathcal{N}^{(i)} \in \mathbb{R}^{d_i \times 1}$ as it should be since γ^i is scalar (a single parameter to be perturbed which affects to the set of d_i responses in $\boldsymbol{x}^i$).

Dependence on $\boldsymbol{b}^i$: Proof of Eq. 31. Before deriving with regard to $\boldsymbol{b}^i$, it is convenient to rearrange the terms in Eq. 10 to put the only parameter depending on $\boldsymbol{b}^i$ (the denominator) at the end. As the Hadamard product is commutative we can rearrange terms, and using the diagonal matrix notation for the terms independent of $\boldsymbol{b}^i$,

$$\mathcal{N}^{(i)} = \mathrm{sign}(\boldsymbol{y}^i) \odot \boldsymbol{e^i} \odot \frac{1}{\mathcal{D}^{(i)}(\boldsymbol{e}^i)} = \mathbb{D}_{\mathrm{sign}(\boldsymbol{y}^i)} \cdot \mathbb{D}_{\boldsymbol{e}^i} \cdot \frac{1}{\mathcal{D}^{(i)}(\boldsymbol{e}^i)} \tag{S4.6}$$

Now, we derive the vector at the end (the Hadamard quotient with the denominator),

$$\nabla_{\boldsymbol{b}^i}\mathcal{N}^{(i)} = \mathbb{D}_{\mathrm{sign}(\boldsymbol{y}^i)} \cdot \mathbb{D}_{\boldsymbol{e}^i} \cdot \frac{\partial (\boldsymbol{b}^i + H^i \cdot \boldsymbol{e}^i)^{-1}}{\partial \boldsymbol{b}^i} = -\mathbb{D}_{\mathrm{sign}(\boldsymbol{y}^i)} \cdot \mathbb{D}_{\boldsymbol{e}^i} \cdot \mathbb{D}^{-2}_{\mathcal{D}^{(i)}(\boldsymbol{e}^i)} \cdot \mathbb{1} \tag{S4.7}$$

which is Eq. 31. This Jacobian is a $d_i \times d_i$ matrix consistently with the dimensions of the output and of the vector $\boldsymbol{b}^i$.

Dependence on general H^i: Proof of Eq. 32. With the same rearrangement done in Eq. S4.6 to leave the denominator in the end, we take derivatives with regard to H^i,

$$\nabla_{H^i}\mathcal{N}^{(i)} = \mathbb{D}_{\mathrm{sign}(\boldsymbol{y}^i)}\cdot\mathbb{D}_{\boldsymbol{e}^i}\cdot\frac{\partial(\boldsymbol{b}^i + H^i\cdot\boldsymbol{e}^i)^{-1}}{\partial H^i} = -\mathbb{D}_{\mathrm{sign}(\boldsymbol{y}^i)}\cdot\mathbb{D}_{\boldsymbol{e}^i}\cdot\mathbb{D}^{-2}_{\mathcal{D}^{(i)}(\boldsymbol{e}^i)}\cdot\frac{\partial H^i\cdot\boldsymbol{e}^i}{\partial H^i} \quad \text{(S4.8)}$$

where, again, we apply the block diagonal result for the derivative of a linear function with regard to the parameters, Eq. S2.4, leading to $\mathbb{B}^{d_i}_{\boldsymbol{e}^{i\top}}$ and hence obtaining Eq. 32. Given this final block-diagonal term this Jacobian is a $d_i \times (d_i \times d_i)$ matrix, consistently with the number of parameters than may vary in H^i. Note also that small perturbations of the response from perturbations in H^i should be computed using is the column-wise rearrangement for H^i defined in Eq. S2.2.

Dependence on parametric kernels: Proof of Eqs. 35 and 36. Considering the Gaussian parametrization, Eq. 33 in the main text, the derivative with regard to the width of the k-th row is,

$$\frac{\partial\mathcal{N}^{(i)}}{\partial\sigma^i_k} = \frac{\partial\mathcal{N}^{(i)}}{\partial H^i}\cdot\frac{\partial H^i}{\partial H^i_{k\star}}\cdot\frac{\partial H^i_{k\star}}{\partial\sigma^i_k} \quad \text{(S4.9)}$$

The matrix that transmits the variations in a single row of the kernel into the whole vector-rearranged kernel, $\Delta\,\mathrm{vect}({H^i}^\top) = \frac{\partial H^i}{\partial H^i_{k\star}}\cdot\Delta {H^i_{k\star}}^\top$, has to be formed by $d_i - 1$ replicas of the zero matrix, with the identity matrix, $\mathbb{1}$, in the k-th location,

$$\frac{\partial H^i}{\partial H^i_{k\star}} = \begin{pmatrix} \emptyset_{d_i\times d_i} \\ \emptyset_{d_i\times d_i} \\ \vdots \\ \emptyset_{d_i\times d_i} \\ \mathbb{1}_{d_i\times d_i} \\ \emptyset_{d_i\times d_i} \\ \vdots \\ \emptyset_{d_i\times d_i} \end{pmatrix} \begin{matrix} \text{1st submatrix} \\ \text{2nd submatrix} \\ \vdots \\ \\ k\text{-th submatrix} \\ \\ \vdots \\ d_i\text{-th submatrix} \end{matrix} \quad \text{(S4.10)}$$

This derivative, $\frac{\partial H^i}{\partial H^i_{k\star}}$, selects a single submatrix in the huge block-diagonal matrix, $\mathbb{B}^{d_i}_{\boldsymbol{e}^i}$, that is in $\frac{\partial\mathcal{N}^{(i)}}{\partial H^i}$, Eq. 32. As a result, this product leads to a square matrix where the only nonzero row is in the k-th location,

$$\mathbb{B}^{d_i}_{\boldsymbol{e}^i}\cdot\frac{\partial H^i}{\partial H^i_{k\star}} = \begin{pmatrix} \emptyset_{1\times d_i} \\ \emptyset_{1\times d_i} \\ \vdots \\ \emptyset_{1\times d_i} \\ {\boldsymbol{e}^i}^\top \\ \emptyset_{1\times d_i} \\ \vdots \\ \emptyset_{1\times d_i} \end{pmatrix} \begin{matrix} \text{1st row} \\ \text{2nd row} \\ \vdots \\ \\ k\text{-th row} \\ \\ \vdots \\ d_i\text{-th row} \end{matrix} \quad \text{(S4.11)}$$

When multiplying the above matrix by the extra diagonal matrix in $\frac{\partial\mathcal{N}^{(i)}}{\partial H^i}$, Eq. 32, we have the only nonzero row scaled by the k-th component of the diagonal of $-\mathbb{D}_{\mathrm{sign}(\boldsymbol{y}^i)}\cdot$

$\mathbb{D}_{\boldsymbol{e}^i} \cdot \mathbb{D}^{-2}_{\mathcal{D}^{(i)}(\boldsymbol{e}^i)}$, i.e.

$$\frac{\partial \mathcal{N}^{(i)}}{\partial \sigma^i_k} = \begin{pmatrix} \emptyset_{1\times d_i} \\ \emptyset_{1\times d_i} \\ \vdots \\ \emptyset_{1\times d_i} \\ -\text{sign}(y^i_k)\; e^i_k\; {\mathcal{D}^{(i)}}^{-2}_k \quad {\boldsymbol{e}^i}^\top \\ \emptyset_{1\times d_i} \\ \vdots \\ \emptyset_{1\times d_i} \end{pmatrix} \cdot \frac{\partial H^i_{k\star}}{\partial \sigma^i_k} \tag{S4.12}$$

Now, taking into account the straightforward derivative of a normalized 2D Gaussian with regard to its width,

$$\frac{\partial}{\partial \sigma}\left[c\frac{dp^2}{2\pi\,\sigma^2}\,\exp\left(-\frac{\Delta^2_{kk'}}{2\,\sigma^2}\right)\right] \;=\; c\frac{dp^2}{2\pi\,\sigma^5}\left(\Delta^2_{kk'} - 2\,\sigma^2\right)\,\exp\left(-\frac{\Delta^2_{kk'}}{2\,\sigma^2}\right)$$

and considering that this holds $\forall k' \in \text{subband}\, k$, the considered $\frac{\partial H^i_{k\star}}{\partial \sigma^i_k}$ is a column vector:

$$\nabla_{\sigma^i_k} \mathcal{N}^{(i)} = \begin{pmatrix} \emptyset_{1\times d_i} \\ \emptyset_{1\times d_i} \\ \vdots \\ \emptyset_{1\times d_i} \\ -\text{sign}(y^i_k)\; e^i_k\; {\mathcal{D}^{(i)}}^{-2}_k \quad {\boldsymbol{e}^i}^\top \\ \emptyset_{1\times d_i} \\ \vdots \\ \emptyset_{1\times d_i} \end{pmatrix} \cdot \begin{pmatrix} F^i_{k1} \\ F^i_{k2} \\ F^i_{k3} \\ \\ \vdots \\ \\ F^i_{kd_i} \end{pmatrix} \tag{S4.13}$$

where

$$F^i_{kk'} = \begin{cases} 0 & \forall\; k' \notin \text{subband}\;\; k \\ \\ c^i_k\, \frac{dp_{k1} dp_{k2}}{2\pi\;{\sigma^i_k}^5}\; \left(\Delta^2_{kk'} - 2\,{\sigma^i_k}^2\right)\; e^{-\frac{\Delta^2_{kk'}}{2\;{\sigma^i_k}^2}} & \forall\; k' \in \text{subband}\;\; k \end{cases}$$

Note that the matrix-on-vector product in Eq. S4.13 is a vector with a single nonzero element (the one in the k-th location). Therefore, the derivatives with regard to all the widths can be expressed in a single matrix expression by replicating the transposed vector ${\boldsymbol{e}^i}^\top$ in d_i rows, and by stacking the different column vectors $F^i_{kk'}$ $\forall\, k$. Then, the elements we are looking for are in the diagonal of the resulting matrix. This leads to the diagonal matrix in Eq. 35.

In the case of the derivative with regard to the amplitudes of the Gaussians, Eq. S4.12 also holds $\forall\, k$ with the corresponding change of variables. Then, taking into account that the remaining derivative with regard to the amplitude is simply the Gaussian, and the consideration of all coefficients k stacked leads to Eq. 36.
