# Supplementary material for: Derivatives and inverse of cascaded linear+nonlinear neural models
Source: PLoS One. 2018 Oct 15;13(10):e0201326. doi: 10.1371/journal.pone.0201326 (PMC6188639; doi:10.1371/journal.pone.0201326)
Supplement: S5 File — (PDF) [file pone.0201326.s005.pdf]

Supporting Information file S5:

S5. Derivation of the inverse

Here we derive the inverse of the Divisive Normalization (Eq. 39 in the main text).

From Eq. 10 of the main text, the vector of absolute values of the responses is $|\boldsymbol{x}^i| = \mathbb{D}^{-1}_{\left(\boldsymbol{b}^i + H^i \cdot \boldsymbol{e}^i\right)} \cdot \boldsymbol{e}^i$. Therefore, inverting the matrix, it holds,

$$\mathbb{D}_{\left(\boldsymbol{b}^i + H^i \cdot \boldsymbol{e}^i\right)} \cdot |\boldsymbol{x}^i| = \boldsymbol{e}^i$$

which can be written using the Hadamard product,

$$(\boldsymbol{b}^i + H^i \cdot \boldsymbol{e}^i) \odot |\boldsymbol{x}^i| = \boldsymbol{e}^i$$

Now, using the diagonal matrix notation of Hadamard products and the fact that $\mathbb{D}_{\boldsymbol{a}} \cdot \boldsymbol{b} = \mathbb{D}_{\boldsymbol{b}} \cdot \boldsymbol{a}$, we have:

$$\begin{gathered}
\mathbb{D}_{\boldsymbol{b}^i} \cdot |\boldsymbol{x}^i| + \mathbb{D}_{|\boldsymbol{x}^i|} \cdot H^i \cdot \boldsymbol{e}^i = \boldsymbol{e}^i \\
\mathbb{D}_{\boldsymbol{b}^i} \cdot |\boldsymbol{x}^i| = \left(\mathbb{1} - \mathbb{D}_{|\boldsymbol{x}^i|} \cdot H^i\right) \cdot \boldsymbol{e}^i \\
\boldsymbol{e}^i = \left(\mathbb{1} - \mathbb{D}_{|\boldsymbol{x}^i|} \cdot H^i\right)^{-1} \cdot D_{\boldsymbol{b}^i} \cdot |\boldsymbol{x}^i|
\end{gathered}$$

and considering that $\boldsymbol{e}^i = |\boldsymbol{y}^i|^{\gamma^i}$, and that $\boldsymbol{y}^i$ inherits the sign from $\boldsymbol{y}^i$, it follows Eq. 39.
