# Supplementary material for: Derivatives and inverse of cascaded linear+nonlinear neural models
Source: PLoS One. 2018 Oct 15;13(10):e0201326. doi: 10.1371/journal.pone.0201326 (PMC6188639; doi:10.1371/journal.pone.0201326)
Supplement: S6 File — (PDF) [file pone.0201326.s006.pdf]

Supporting Information file S6:

S6. Region-based approach to MAximum Differentiation

Analytic MAximum Differentiation involves computing the eigenvectors of the metric matrix based on the Jacobian (Eq. 23 in the main text). This matrix is huge even for moderate size images and one would like to break this problem into smaller pieces. Here we consider how a region-based strategy may affect MAD.

The use of patch-wise strategies implies assuming that perception of a patch is independent of the content of neighbor patches. Caution has to be taken with such an assumption. In fact, the main reason to include the nonlinear stages is accounting for the masking effect of the neighbor sensors tuned to surrounding locations. The scale of the local interactions between the sensors (e.g. the width of the connections in L^i and the width of the interaction in H^i) will induce edge effects if patch size is limited. Therefore, this is the length scale that must be taken into account in order to break the problem into pieces. For example, in the model used in the Discussion the wider interaction has a length scale of 0.2 degrees (see the specific values in the Toolbox, in the S8 File).

Here, we point out that assuming region-independence in perception implies certain structure in $\nabla_{\boldsymbol{x}^0} S$. This structure has consequences on the metric matrix, which leads to simplified solutions of MAD, which are also consistent with the original region-independence assumption and with the quadratic summation assumption.

Imagine that large images are formed by N distinct spatial blocks. In this case, data corresponding to the difference between two images can be arranged in a column vector by stacking the vectors corresponding to the N distinct regions: $\Delta\boldsymbol{x}^0 = \left(\Delta\boldsymbol{x}^0_{[1]}{}^\top \, \Delta\boldsymbol{x}^0_{[2]}{}^\top \cdots \Delta\boldsymbol{x}^0_{[N]}{}^\top\right)^\top$. Assuming that these distinct regions are perceptually independent, implies a block-diagonal structure in $\nabla_{\boldsymbol{x}^0} S$, because the variation of the responses corresponding to the i-th region should not depend on the variation of the inputs for the j-th region:

$$\begin{pmatrix} \Delta\boldsymbol{x}^n_{[1]} \\ \Delta\boldsymbol{x}^n_{[2]} \\ \vdots \\ \Delta\boldsymbol{x}^n_{[N]} \end{pmatrix} = \begin{pmatrix} \nabla_{\boldsymbol{x}^0_{[1]}} S(\boldsymbol{x}^0_{[1]}) & \emptyset_{d_n\times d_0} & \cdots & \emptyset_{d_n\times d_0} \\ \emptyset_{d_n\times d_0} & \nabla_{\boldsymbol{x}^0_{[2]}} S(\boldsymbol{x}^0_{[2]}) & \cdots & \emptyset_{d_n\times d_0} \\ \vdots & \vdots & \ddots & \vdots \\ \emptyset_{d_n\times d_0} & \emptyset_{d_n\times d_0} & \cdots & \nabla_{\boldsymbol{x}^0_{[N]}} S(\boldsymbol{x}^0_{[N]}) \end{pmatrix} \cdot \begin{pmatrix} \Delta\boldsymbol{x}^0_{[1]} \\ \Delta\boldsymbol{x}^0_{[2]} \\ \vdots \\ \Delta\boldsymbol{x}^0_{[N]} \end{pmatrix} \tag{S6.1}$$

where each (relatively small) rectangular sub-matrix, $\nabla_{\boldsymbol{x}^0_{[i]}} S(\boldsymbol{x}^0_{[i]})$, describes the behavior for the i-th region.

In this situation, in the 2nd-order (or local-linear) approximation, the perceptual difference induced by the large $\Delta\boldsymbol{x}^0$ would be given by $\mathbb{d}_p^2 = \Delta\boldsymbol{x}^0{}^\top \cdot \nabla_{\boldsymbol{x}^0} S(\boldsymbol{x}^0)^\top \cdot \nabla_{\boldsymbol{x}^0} S(\boldsymbol{x}^0) \cdot \Delta\boldsymbol{x}^0$, and using the block diagonal structure in Eq. S6.1, one has:

$$\mathbb{d}_p^2 = \left(\Delta\boldsymbol{x}^0_{[1]}{}^\top \, \Delta\boldsymbol{x}^0_{[2]}{}^\top \cdots \Delta\boldsymbol{x}^0_{[2]}{}^\top\right) \cdot \begin{pmatrix} M(\boldsymbol{x}^0_{[1]}) & \emptyset_{d_0\times d_0} & \cdots & \emptyset_{d_0\times d_0} \\ \emptyset_{d_0\times d_0} & M(\boldsymbol{x}^0_{[2]}) & \cdots & \emptyset_{d_0\times d_0} \\ \vdots & \vdots & \ddots & \vdots \\ \emptyset_{d_0\times d_0} & \emptyset_{d_0\times d_0} & \cdots & M(\boldsymbol{x}^0_{[N]}) \end{pmatrix} \cdot \begin{pmatrix} \Delta\boldsymbol{x}^0_{[1]} \\ \Delta\boldsymbol{x}^0_{[2]} \\ \vdots \\ \Delta\boldsymbol{x}^0_{[N]} \end{pmatrix} \tag{S6.2}$$

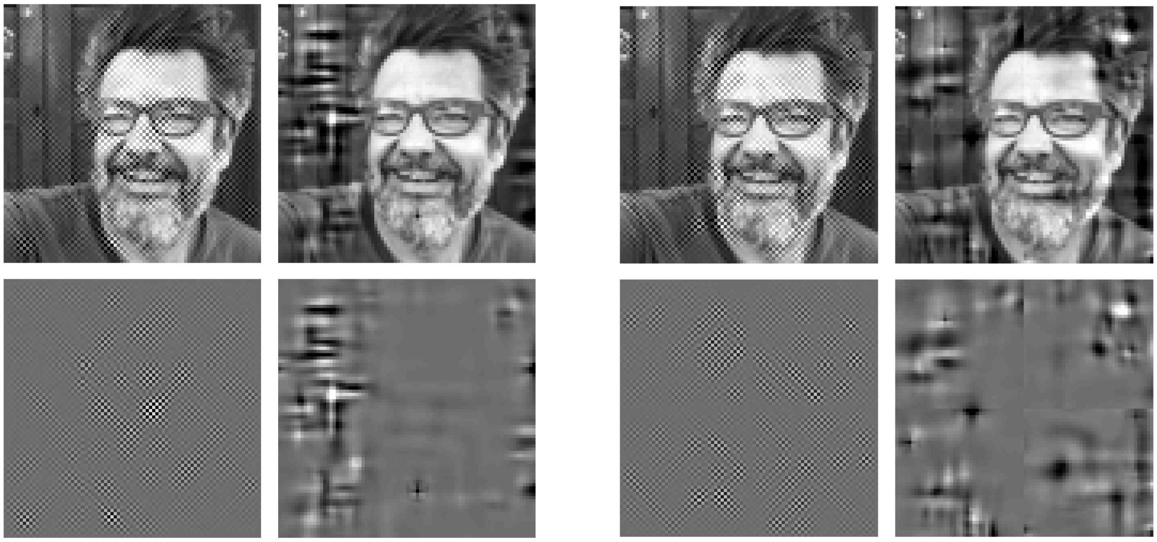

Fig S6.1. **Global vs region-based MAD computation.** *Left:* best and worst MAD-analytic images computed from an image subtending 1.25 deg. *Right:* best and worst MAD-analytic images computed from 4 subimages subtending 0.63 deg each.

where each (relatively small) square sub-matrix $M(\boldsymbol{x}^0_{[i]}) = \nabla_{\boldsymbol{x}^0_{[i]}} S(\boldsymbol{x}^0_{[i]})^\top \cdot \nabla_{\boldsymbol{x}^0_{[i]}} S(\boldsymbol{x}^0_{[i]})$ is the metric matrix corresponding to the i-th region.

Eq. S6.2 has one interesting consequence for its use in Maximum Differentiation: since the eigenvectors of a (large) block-diagonal matrix can be computed from the eigenvectors of the (smaller) blocks in the diagonal [1], there is no need to explicitly build and work with the huge matrices in Eqs. S6.1 and S6.2. This relieving mathematical result is completely consistent with intuition: if perceptual independence is assumed (and responses are computed block-wise), then, large MAD images can be also computed block-wise from the eigenvectors of the smaller metric matrices corresponding to each image block.

Fig S6.1 shows an example of the comparison of block-wise versus global approaches to compute MAD images. In both best and worst images the energy is focused roughly in the same spatial regions with the same frequency content. Qualitative behavior is similar meaning that edge effects are negligible. The above warning about assuming region-independence in perception implies that, strictly speaking, one cannot ensure that patch-wise computation of eigendistortions will not modify the judgement and the interpretation of MAD. However, as suggested by the example in Fig S6.1, the response to the MAD question is fairly independent of block size given *big-enough* regions. How big is this is an experimental issue that would probably depend on the specific length-scale of the parameter that one is addressing in the MAD experiment. Qualitative check of edge effects at different sizes may be necessary before launching the actual experiment to choose a small, yet safe, block size.

A lateral consequence of Eq. S6.2 is related to the summation of distortions across the visual field. Note that the total distortion is the quadratic sum of individual distortions in the regions of the image, $\mathbb{d}^2_p = \sum_{i=1}^N \Delta\boldsymbol{x}^{0\,\top}_{[i]} \cdot M(\boldsymbol{x}^0_{[i]}) \cdot \Delta\boldsymbol{x}^0_{[i]} = \sum_{i=1}^N \mathbb{d}^{[i]\,2}_p$. This quadratic summation is consistent with the quadratic norm chosen for the summation over individual response elements.

References

1. Golub GH, Van Loan CF. Matrix Computations (3rd Ed.). Baltimore, MD, USA: Johns Hopkins University Press; 1996.
