# Supplementary material for: Derivatives and inverse of cascaded linear+nonlinear neural models
Source: PLoS One. 2018 Oct 15;13(10):e0201326. doi: 10.1371/journal.pone.0201326 (PMC6188639; doi:10.1371/journal.pone.0201326)
Supplement: S7 File — (PDF) [file pone.0201326.s007.pdf]

Supporting Information file S7:

S7. Maximization of correlation with subjective opinion

Here we derive the expression of the derivative of the Pearson correlation between subjective distances and the distances predicted by a perception model with regard to the parameters of the model (Eq. 48 in the main text).

The linear correlation between the ground truth, $\boldsymbol{M}$ and the model predictions $\boldsymbol{D}$ is,

$$\varrho = \frac{E((\boldsymbol{M} - \bar{\boldsymbol{M}})(\boldsymbol{D} - \bar{\boldsymbol{D}}))}{\sigma(\boldsymbol{M})\sigma(\boldsymbol{D})} \tag{S7.1}$$

where $E(\cdot)$ stands for expected value, $\bar{\boldsymbol{v}}$ stands for the average of vector $\boldsymbol{v}$, and $\sigma(\cdot)$ stands for standard deviation.

However, there is a more convenient expression to apply the chain rule in the derivatives with regard to the parameters. Note that the sums in the average to compute $E(\cdot)$ and $\sigma(\cdot)$ can be written as dot products and norms, and subtraction of the mean can be written as $\boldsymbol{M_s} = \boldsymbol{M} - \frac{1}{N} \cdot \mathbb{1} \cdot \boldsymbol{M}$, where $\mathbb{1}$ is an all-ones matrix. As a result, the linear correlation can be written as,

$$\varrho = \frac{\boldsymbol{M_s}^T \cdot \boldsymbol{D_s}}{|\boldsymbol{M_s}||\boldsymbol{D_s}|} \tag{S7.2}$$

In this way, the derivatives of $\varrho(\boldsymbol{\Theta})$ with regard to $\boldsymbol{\Theta}$:

$$\frac{d\varrho}{d\boldsymbol{\Theta}} = \underbrace{\frac{d\varrho}{d\boldsymbol{D_s}}}_{(1)} \cdot \underbrace{\frac{d\boldsymbol{D_s}}{d\boldsymbol{\Theta}}}_{(2)} \qquad \text{where} \begin{cases} \frac{d\varrho}{d\boldsymbol{D_s}} \in \mathbb{R}^{1\times N} \\ \frac{d\boldsymbol{D_s}}{d\boldsymbol{\Theta}} \in \mathbb{R}^{N\times m} \end{cases} \tag{S7.3}$$

Developing the term (1)

$$\begin{aligned} \frac{d\varrho}{d\boldsymbol{D_s}} =& \frac{d}{d\boldsymbol{D_s}}\left[\frac{\boldsymbol{M_s}^T\boldsymbol{D_s}}{|\boldsymbol{M_s}|\cdot|\boldsymbol{D_s}|}\right] \\ =& \frac{\boldsymbol{M_s}^T}{|\boldsymbol{M_s}|\cdot|\boldsymbol{D_s}|} + \frac{\boldsymbol{M_s}^T\boldsymbol{D_s}}{|\boldsymbol{M_s}|}\cdot\frac{d}{d\boldsymbol{D_s}}(\boldsymbol{D_s}^T\cdot\boldsymbol{D_s})^{-1/2} \\ =& \frac{\boldsymbol{M_s}^T}{|\boldsymbol{M_s}|\cdot|\boldsymbol{D_s}|} - \frac{1}{2}\frac{\boldsymbol{M_s}^T\boldsymbol{D_s}}{|\boldsymbol{M_s}|}\cdot(\boldsymbol{D_s}^T\cdot\boldsymbol{D_s})^{-3/2}\cdot\frac{d}{d\boldsymbol{D_s}}(\boldsymbol{D_s}^T\cdot\boldsymbol{D_s}) \\ =& \frac{\boldsymbol{M_s}^T}{|\boldsymbol{M_s}|\cdot|\boldsymbol{D_s}|} - \frac{\boldsymbol{M_s}^T\boldsymbol{D_s}}{|\boldsymbol{M_s}|}\cdot\frac{1}{|\boldsymbol{D_s}|^3}\cdot\boldsymbol{D_s}^T \\ =& \frac{\boldsymbol{M_s}^T}{|\boldsymbol{M_s}|\cdot|\boldsymbol{D_s}|} - \frac{\boldsymbol{M_s}^T\boldsymbol{D_s}}{|\boldsymbol{M_s}|\cdot|\boldsymbol{D_s}|^3}\cdot\boldsymbol{D_s}^T \end{aligned} \tag{S7.4}$$

Developing the term (2)

$$\frac{d\boldsymbol{D_s}}{d\boldsymbol{\Theta}} = \frac{d}{d\boldsymbol{\Theta}}\left(\boldsymbol{D} - \frac{1}{N}\mathbb{1}\boldsymbol{D}\right) = \frac{d\boldsymbol{D}}{d\boldsymbol{\Theta}} - \frac{1}{N}\mathbb{1}\frac{d\boldsymbol{D}}{d\boldsymbol{\Theta}} \tag{S7.5}$$

where,

$$\frac{d\boldsymbol{D}}{d\boldsymbol{\Theta}} = \begin{pmatrix} \frac{\partial \mathbb{d}_p^{[1]}}{\partial \boldsymbol{\Theta}} \\ \frac{\partial \mathbb{d}_p^{[2]}}{\partial \boldsymbol{\Theta}} \\ \vdots \\ \frac{\partial \mathbb{d}_p^{[N]}}{\partial \boldsymbol{\Theta}} \end{pmatrix},$$

where, for the i-th image,

$$\begin{aligned}
\frac{\partial \mathbb{d}_p^{[i]}}{\partial \boldsymbol{\Theta}} &= \frac{\partial \left(\Delta \boldsymbol{x}_{[i]}^{n\top} \cdot \Delta \boldsymbol{x}_{[i]}^{n}\right)^{1/2}}{\partial \boldsymbol{\Theta}} = \frac{1}{2\mathbb{d}_p^{[i]}} \cdot \frac{\partial \left(\Delta \boldsymbol{x}_{[i]}^{n\top} \cdot \Delta \boldsymbol{x}_{[i]}^{n}\right)}{\partial \boldsymbol{\Theta}} \\
&= \frac{1}{2\mathbb{d}_p^{[i]}} 2 \cdot \Delta \boldsymbol{x}_{[i]}^{n\top} \cdot \frac{\partial \Delta \boldsymbol{x}_{[i]}^{n}}{\partial \boldsymbol{\Theta}} \\
&= \frac{1}{\mathbb{d}_p^{[i]}} \cdot \Delta \boldsymbol{x}_{[i]}^{n\top} \cdot \frac{\partial (\boldsymbol{z}_{[i]}^{n} - \boldsymbol{x}_{[i]}^{n})}{\partial \boldsymbol{\Theta}} \\
&= \frac{1}{\mathbb{d}_p^{[i]}} \cdot \Delta \boldsymbol{x}_{[i]}^{n\top} \cdot \left(\frac{\partial \boldsymbol{z}_{[i]}^{n}}{\partial \boldsymbol{\Theta}} - \frac{\partial \boldsymbol{x}_{[i]}^{n}}{\partial \boldsymbol{\Theta}}\right) \\
&= \frac{1}{\mathbb{d}_p^{[i]}} \cdot \Delta \boldsymbol{x}_{[i]}^{n\top} \cdot \left[\nabla_{\boldsymbol{\Theta}} S(\boldsymbol{z}_{[i]}^{0}) - \nabla_{\boldsymbol{\Theta}} S(\boldsymbol{x}_{[i]}^{0})\right]
\end{aligned} \tag{S7.6}$$

Plugging Eqs. S7.4-S7.6 into Eq. S7.3, one gets Eq. 48.
