# Supplementary material for: Derivatives and inverse of cascaded linear+nonlinear neural models
Source: PLoS One. 2018 Oct 15;13(10):e0201326. doi: 10.1371/journal.pone.0201326 (PMC6188639; doi:10.1371/journal.pone.0201326)
Supplement: S8 File — (PDF) [file pone.0201326.s008.pdf]

Supporting Information file S8:

S8. The BioMultiLayer-L+NL Toolbox

This Matlab toolbox implements all the maths related to the kind of multilayer feedforward L+NL neural models considered in the paper. Specifically, it implements the vision model detailed in the S1 File, which consists of a casacade of isomorphic linear+nonlinear transforms based on linear receptive fields and canonical Divisive Normalization nonlinearities).

The BioMultiLayer-L+NL toolbox includes the forward transform, the inverse, and all the derivatives (w.r.t. the signal and w.r.t. the parameters). The derivatives allow (i) Novel MAximum Differentiation (MAD) psychophysics and (ii) fitting the model from classical psychophysics. The inverse allows improved decoding of neural signals.

For convenience, this release includes other public-domain toolboxes (please cite these sources as well):

- CSF of the Standard Spatial Observer (SSO folder) by J. Malo and A.B. Watson [1].
- Steerable wavelet pyramid (matlabPyrTools folder) by E.P. Simoncelli [2].

S8.1 Installing the toolbox

(1) Download the toolbox from http://isp.uv.es/docs/BioMultiLayer_L_NL.zip. (2) Decompress all the contents of the file. (3) Compile the mex files of the matlabPyrTools toolbox. (4) Include the folders in the Matlab path.

Once the toolbox is installed you may enter `help BioMultiLayer_L_NL` at the Matlab prompt to get an overview of all the routines in the toolbox. It is convenient to take a look at the demo scripts included in the toolbox for worked-out examples.

S8.2 Basic routines: responses, derivatives and inverse

The basic use of the BioMultiLayer-L+NL toolbox reduces to two routines (the forward and the inverse transform):

- Given an image, deep_model_DN_isomorph.m computes the responses and the Jacobians of the vision model made of isomorphic Linear+Nonlinear layers with Divisive Normalization. This function sequentially calls stage_L_NL.m that computes the response and the Jacobians of a single layer in the network.
- Given a response vector, inv_deep_model_DN_isomorph.m decodes the response and reconstructs the input image. This function sequentially calls the function inv_stage_L_NL.m that computes the inverse of a single layer in the network.

The user is referred to the help of these functions for details on how to use them. Additionally, step-by-step examples on how to use the above functions is given in the demo script demo_deep_DN_iso.m. Particularly relevant is how to select which Jacobian(s) should be computed. This is controlled with a structure described in the help of stage_L_NL.m.

Initialization note: before using the above utilities, (a) images have to be prepared for the toolbox, and (b) the parameters of the model have to be set.

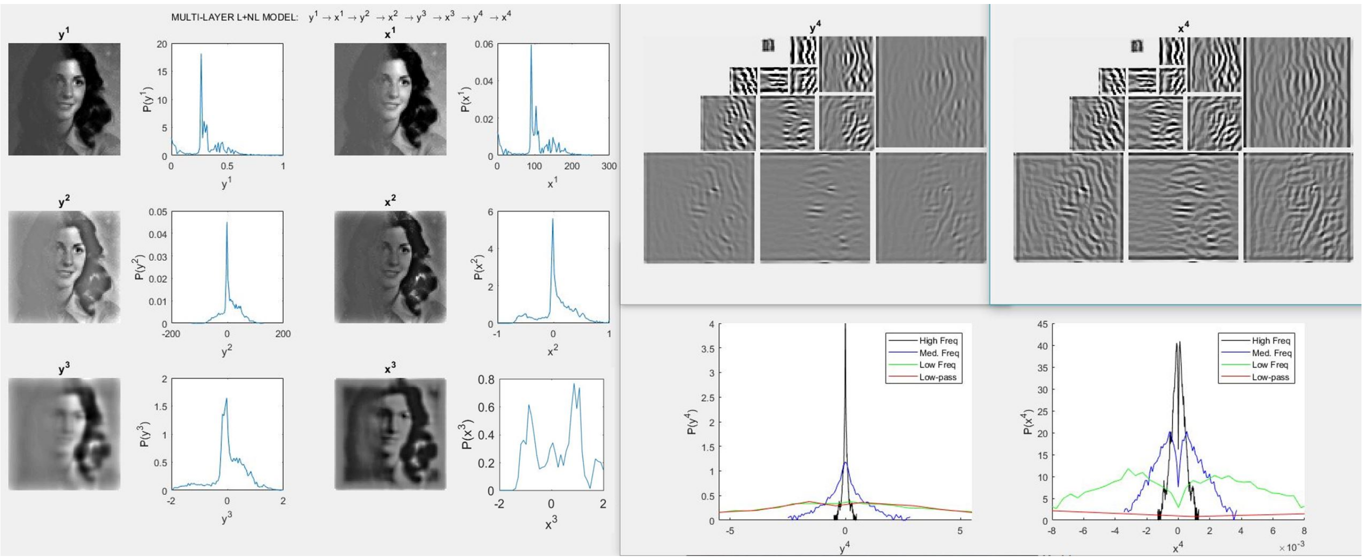

Fig S8.1. **Forward Transform (in the demo script** `demo_deep_DN_iso.m`**).** Responses and marginal PDFs along the layers of the network. Note that the bimodal PDFs of $\boldsymbol{x}^3$ and $\boldsymbol{x}^4$ are consistent with the predictive behavior reported in [3]. Equalization behavior at $\boldsymbol{x}^1$ reported at [4] is not that evident in this example because this is a low-dynamic-range image.

(a) Input stimuli, image normalization, and image arrangement The current version of the `BioMultiLayer-L+NL` toolbox operates on *achromatic images* in normalized luminance units. This means that in the current implementation the model starts from luminance vectors, $\boldsymbol{y}^0$, and not from hyperspectral stimuli, $\boldsymbol{x}^0$. In other words, the spectral and the chromatic elements of the first stage in Fig 1 of the main text, or the linear integration of the spectrum, L^1, in Eq. S1.1, are not included in this release. This missing linear stage, L^1, can be easily implemented using the `Colorlab` toolbox [5].

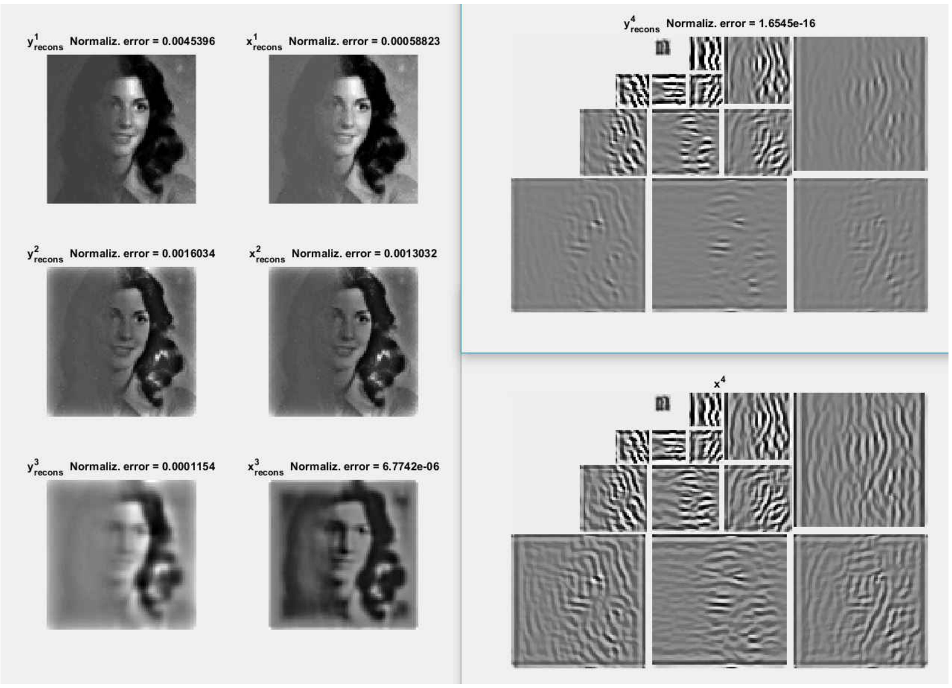

Fig S8.2. **Inverse Transform (in the demo script** `demo_deep_DN_iso.m`**).** Decoded signals from the response at the last layer of the network.

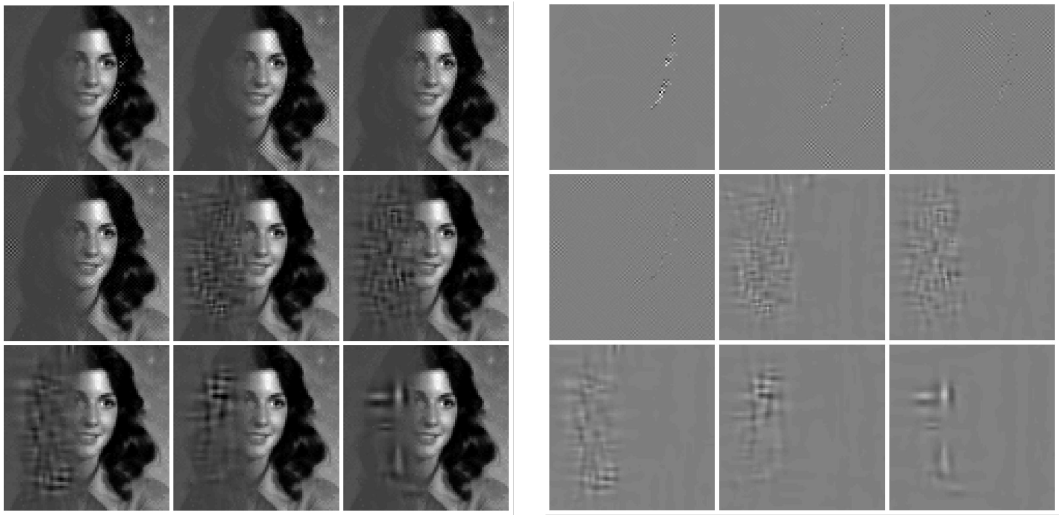

Fig S8.3. Derivatives w.r.t. the signal: Analytic MAD (in the demo script `demo_deep_DN_iso.m`) Left panel: Distorted images in the directions of low and high eigenvalues of the 2nd order metric matrix. Low eigendistortions correspond to the upper-left images where distortions are not noticeable, and high eigendistortions correspond to the lower-right images where distortions are highly noticeable. Right panel: isolated distortions. All the distortions have the same energy (induce the same MSE), but they are visually very different. Note that highly visible distortions are concentrated in the low contrast part of the image (and the other way around for highly visible distortions), which is consistent with the masking phenomenon and indicates the quality of the model.

Specifically, the `Colorlab` functions `spect2tri.m` and `xyz2atd.m` compute CIE XYZ tristimulus values from radiances, and transform the CIE XYZ result to convenient opponent chromatic representations (luminance, red-green and yellow-blue) [6, 7].

Normalized luminance refers to division by *what is assumed to be the maximum luminance in the considered class of scenes.* This implies that input values in $\boldsymbol{y}^0$ are mainly in the [0, 1] range except for highlights that may be over 1. See the comment on proper image normalization and luminance calibration in `deep_model_DN_isomorph.m`. More accurate transforms from conventional digital images to tristimulus images can be done using the `Colorlab` [5] or the `Psychtoolbox` [8].

If your input luminance image is a matrix, `y0`, vector arrangement according to the *last-dimension-first* convention cited in Eq. 1 in the main text, is simply obtained using `y0(:)`, and large images can be patch-wise vectorized using `im2col.m`. The `VistaLab` toolbox [9] has convenient generalizations of these vectorization functions to be applied in spatio-spectral (or spatio-temporal) arrays (namely `im2colcube.m` and `col2imcube.m`). These could be applied to extend the current toolbox to start from the hyperspectral stimulus $\boldsymbol{x}^0$ instead of starting from $\boldsymbol{y}^0$.

(b) Model parameters The considered network has many parameters. The Jacobians implemented in this toolbox enable experimental methods to determine the parameters. In fact, a side result of this paper is the specific set of values of the parameters obtained from MAD psychophysics and image quality optimization. These parameters have to be passed to different functions of the `BioMultiLayer-L+NL` toolbox in a specific `struct` variable. In order to simplify the construction of the parameter variable, it is generated in the script `parameters_DN_isomorph.m`. An example on how to set the parameters and call the script is given in `demo_deep_DN_iso.m`. If you are happy with certain set of parameters, you may store them in a `*.mat` file so that you will not need to generate them again.

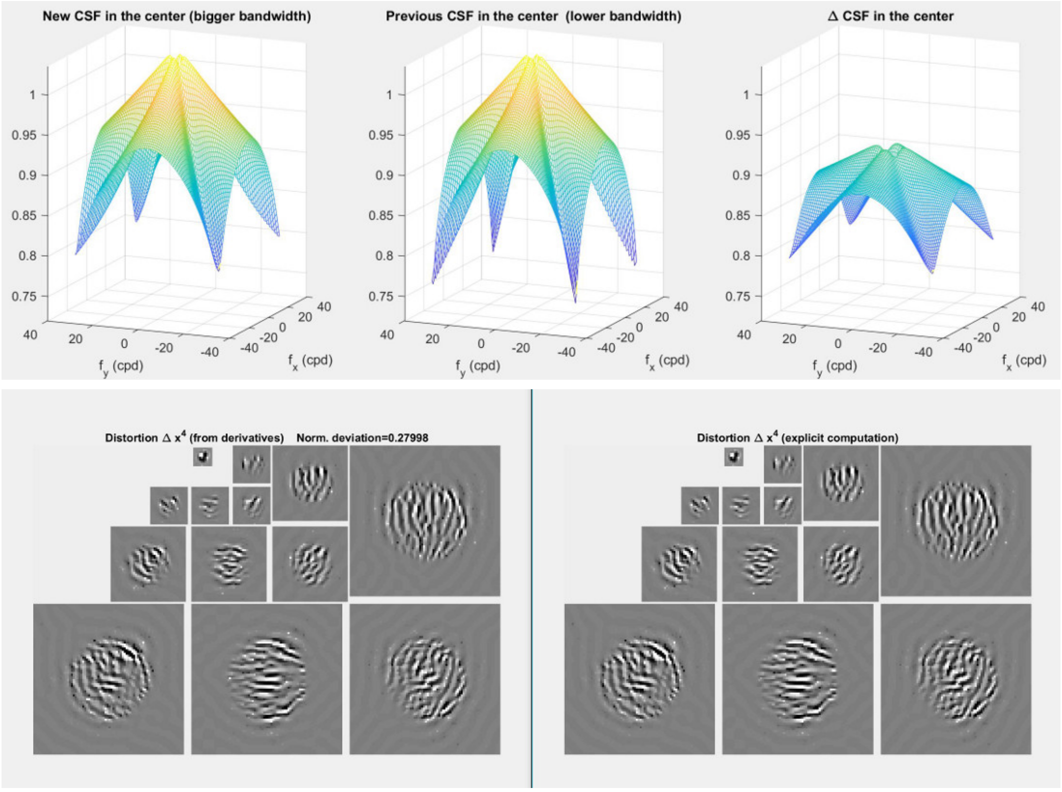

Fig S8.4. Derivatives w.r.t. the parameters: Propagation of the perturbation in the CSF (in the demo script `demo_deep_DN_iso.m`) Imagine a modification in the CSF filter of stage 3. For instance, increasing the bandwidth of the filter. How would it affect the response?. This can be done in two different ways: (1) the exact one (compute the response with and without the modification and subtract), and (2) using the first order approximation based on the Jacobian of the response w.r.t. the parameters (linearly propagate the effect of the modification). The top panel shows the different filter functions used in the center of the visual field, and the bottom panel shows the correponding perturbation in the response computed exactly (right) and according to the first order approximation (left).

S8.3 Advanced routines

The flexible vision model in the `BioMultiLayer-L+NL` toolbox may be used to (a) compute perceptual distances between images, (b) generate stimuli for MAximum Differentiation (MAD), and (c) look for a specific model that maximizes the correlation with subjective opinion in image quality ratings. These advanced applications are available using:

- `metric_deep_DN_iso.m` computes the perceptual distance between an original image and a distorted image according to the multi-layer vision model. The use of this function is illustrated in the script `demo_metric_deep_DN_iso.m`.
- `mad_deep_DN_iso.m` performs the general MAximum Differentiation (MAD) search for an image and a multi-layer vision model defined by its parameters. The use of this function and the equivalent analytic 2nd order result using eigenvectors of the metric is illustrated in the script `demo_mad_DN_iso.m`.
- `maximize_correlation_DN_iso.slurm` launches stochastic gradient descent search in the parameter space to look for the model that maximizes the correlation with the mean opinion score of subjectively rated image quality databases. A `*.slurm` function is invoked because this search is computationally expensive and the optimization has to be parallelized over multiple cores in a cluster. The folder `Corr_max_TID` contains an illustrative example of such parallelization.

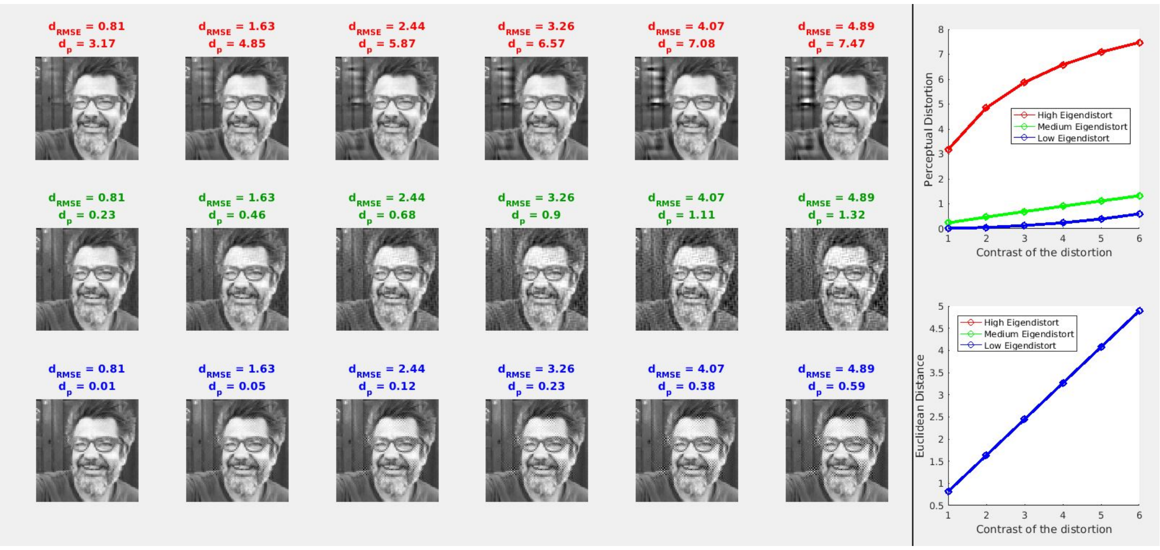

Fig S8.5. **Perceptual distance (demo script** `demo_metric_DN_iso.m`**)** Distortions of different nature (eigen distortions of the metric of low, medium and high eigenvalue in the top, medium and bottom rows). The distortion linearly increases the RMSE contrast from left to right columns. The images in each column have the same RMSE (Euclidean distance) with regard to the original image. The numbers and the plots at the right display the Euclidean and the perceptual distances.

S8.4 Demos

Basic use of the toolbox: `demo_deep_DN_iso.m` This script illustrates the use of the toolbox to compute: (1) the response at the different layers (2) the inverse (decoded signal) (3) the Jacobians (w.r.t. the signal and w.r.t. the parameters).

Responses (see Fig S8.1) and inverses (see Fig S8.2) are shown for an illustrative natural image with nonstationary contrast. Marginal PDFs of the responses are also shown in Fig S8.1 to illustrate the predictive effect of Divisive Normalization. The Jacobian with regard to the stimuli is used to compute the 2nd order metric for that image. We compute eigen-distortions corresponding to such metric matrix and they make perceptual sense (see Fig S8.3). The Jacobian with regard to the parameters is used to estimate the effect of perturbations of the model (see Fig S8.4).

Perceptual distances: `demo_metric_deep_DN_iso.m` This script shows how the Euclidean distance in the response domain is substantially different from the Euclidean distance in the input space, and it is better correlated to subjective opinion. In this script, distortions of different nature are linearly scaled in contrast to lead to different distorted images. Euclidean and perceptual distances are computed and displayed together with the images (see Fig S8.5).

MAximum Differentiation search: `demo_mad_DN_iso.m` This script compares the three methods to generate maximally different images (1) General MAximum Differentiation search (2) Simplified MAximum Differentiation search based on the 2nd order approximation of distance (3) Analytic result based on the 2nd order approximation of distance. The procedure in this demo was used to compute the results in the Discussion section.

Checking the analytical expressions: `check_results.m` This script shows that the analytic results and the implementation are correct.

STAGE 1		STAGE 2		STAGE 3		STAGE 4	
		Error J.sx	1.5e-05 ± 0.3e-05	Error J.sx	1.4e-07 ± 0.4e-07	Error J.sx *	4e-04 ± 10e-04
		Error J.lx	5e-06 ± 1e-06	Error J.lx	2.3e-08 ± 0.4e-08	Error J.lx	5e-07 ± 2e-07
Error J.ny	6e-08 ± 6e-08	Error J.ny	2.9e-05 ± 0.7e-05	Error J.ny	8e-07 ± 5e-07	Error J.ny *	1e-03 ± 3e-03
Error J.scale	2.9e-07 ± 8e-08	Error J.L	6.8e-09 ± 0.7e-09	Error J.L	8e-07 ± 7e-07	Error J.b_full	0.8e-12 ± 3e-12
Error J.b	1e-07 ± 1e-07	Error L_pertur	7e-09 ± 2e-09	Error L_pertur	2e-07 ± 1e-07	Error J.b_subbnd	5e-08 ± 4e-08
Error J.g	3e-08 ± 1e-08	Error L_update	7e-09 ± 2e-09	Error L_update	2e-07 ± 1e-07	Error J.b_scale	8e-08 ± 7e-08
Error J.beta	5e-08 ± 3e-08	Error J.Ls	1.9e-06 ± 0.2e-06	Error J.b	4e-07 ± 1e-07	Error J.b	4e-07 ± 5e-08
		Error J.Lc	7e-08 ± 0.6e-08	Error J.g	3.6e-08 ± 0.3e-08	Error J.g	3e-07 ± 10e-07
		Error J.b	9e-08 ± 1e-08	Error J.H	6e-07 ± 3e-07	Error J.Hs_full *	2e-02 ± 0.2e-02
		Error J.H	2.2e-07 ± 0.2e-07	Error H_pertur	6e-07 ± 4e-07	Error J.Hs_subbnd	1.0e-06 ± 0.3e-06
		Error H_pertur	1.9e-07 ± 0.2e-07	Error H_update	6e-07 ± 3e-07	Error J.Hs_scale	1.1e-06 ± 0.3e-06
		Error H_update	1.9e-07 ± 0.2e-07	Error J.Hs	2.2e-06 ± 0.5e-06	Error J.Hs	1.3e-06 ± 0.4e-06
		Error J.Hs	4.2e-07 ± 0.9e-07	Error J.Hc	9.2e-08 ± 0.1e-08	Error J.Hc_full *	2e-06 ± 1e-06
		Error J.Hc	6.7e-08 ± 0.5e-08			Error J.Hc_subbnd	6e-06 ± 2e-06
						Error J.Hc_scale	6e-06 ± 2e-06
						Error J.Hc	8e-06 ± 2e-06
Error Inv_analyt	4e-12 ± 14e-12	Error Inv_ana_LNL	5.2e-16 ± 0.8e-16	Error Inv_ana_LNL	7e-14 ± 1e-14	Error Inv_exp_LNL	2.2e-06 ± 0.9e-06
Error Inv_expans	5e-03 ± 2e-03	Error Inv_ana_NL	4.0e-16 ± 0.5e-16	Error Inv_ana_NL	9e-16 ± 3e-16	Error Inv_exp_NL	1.2e-16 ± 0.2e-17
		Error Inv_exp_LNL	5.0e-16 ± 0.6e-16	Error Inv_exp_LNL	1e-04 ± 4e-04	Error Inv_ana_LNL	2.3e-06 ± 0.9e-06
		Error Inv_exp_NL	3.0e-16 ± 0.5e-16	Error Inv_exp_NL	1e-04 ± 4e-04	Error Inv_ana_NL	4.9e-16 ± 0.5e-16
		Error Inv_expans	1e-07 ± 7e-07	Error Inv_analyt	2e-11 ± 1e-11	Error Inv_analyt	3e-05 ± 1e-05
		Error inv_analyt	8e-11 ± 40e-11	Error Inv_expans	5e-04 ± 10e-04	Error Inv_expans	1e-03 ± 2e-03

Table S8.1. Numerical check of analytical Jacobians and Inverses. Data displays the normalized error. Normalized error for the Jacobians stands for the ratio of the 2-norm of the numerical-vs-analytical deviation over the 2-norm of the analytical result. For the inverses the normalized error stands for the ratio of the 2-norm of the deviation of the reconstructed signal over the 2-norm of the input signal. The different columns contain the errors at the different stages. The data-blocks from top to bottom are: (1) errors of the derivatives w.r.t. the signal (check of Result I), (2) errors of the derivatives w.r.t. the parameters (check of Result II), and (3) errors of the inverses (isolated in each stage, and propagated through the stages). These numerical experiments were carried out using `check_results.m`. Small errors (errors are always orders of magnitude lower than the actual value) show that expressions and implementation are correct. We highlighted in color the larger errors. Errors of different color indicate different nature in the source of the deviation. Errors in green are obtained at the inverse using the expansion method. As expected, these errors decrease by increasing the number of terms in the expansion (i.e. it is only a matter of increasing the cpu time). Errors in blue come from checking the analytical derivative with finite differences. The finite nature of the increment induces an error. These errors decrease by decreasing the size of the finite difference step. Not a theoretical problem either. Finally, errors in orange correspond to the case where too small increments lead to noisy variations of functions (imagine the differences induced in a Gaussian kernel due to a very small difference in its width). This introduces extra errors that accumulate when considering huge kernels (as is the case in the 4th stage). These deviations reduce when reducing the size of the finite difference in the numerical derivative.

Analytic derivatives are compared to derivatives computed through finite differences. The analytic inverses and the inverses based on expansions are compared to the actual input. Derivatives and inverses are computed using patches from natural images (new patches are randomly selected in each realization of this script). Reasonable values for the parameters of the model are assumed. Agreement between the compared quantities is graphically and numerically assessed. *Graphic assessment:* Values of the analytic results are plotted versus the equivalent numerical results. Good alignment along the unit-slope diagonal means good agreement. *Numeric assessment:* Deviations between the compared results are expressed as ratios between the norm of the deviation over the norm of the analytic result. small values of this ratio mean good agreement.

A summary of the numeric results computed on 50 patches of natural images is given in Table S8.1.

References

1. Watson AB, Malo J. Video quality measures based on the standard spatial observer. Proc. IEEE Int. Conf Im. Proc. 2002. vol. 3: 41–44.
2. Simoncelli EP, Freeman WT, Adelson EH, Heeger DJ. Shiftable multi-scale transforms. IEEE Trans Information Theory. 1992;38(2):587–607. doi:10.1109/18.119725.
3. Malo J, Laparra V. Psychophysically tuned divisive normalization approximately factorizes the PDF of natural images. Neural computation. 2010;22(12):3179–3206.
4. Kane D, Bertalmio M. System gamma as a function of image-and monitor-dynamic range. Journal of Vision. 2016;16(6):4–4.
5. Malo J, Luque MJ. ColorLab: The Matlab toolbox for Colorimetry and Color Vision; 2002. `http://isp.uv.es/code/visioncolor/colorlab.html`.

6. Stockman A, Brainard DH. Color vision mechanisms. In: Bass M, editor. OSA Handbook of Optics (3rd. Ed.). NY: McGraw-Hill; 2010. p. 147–152.

7. Fairchild MD. Color appearance models. Wiley; 2013.

8. Kleiner M, Brainard D, Pelli D, Broussard C, Wolf T, Niehorster D. Psychtoolbox: A Matlab Toolbox for Vision and Neuroscience Research; 1995. http://psychtoolbox.org/.

9. Malo J, Gutiérrez J. VistaLab: The Matlab toolbox for spatio-temporal Vision Models; 2014. http://isp.uv.es/code/visioncolor/vistalab.html.
