# Supplementary material for: Derivatives and inverse of cascaded linear+nonlinear neural models
Source: PLoS One. 2018 Oct 15;13(10):e0201326. doi: 10.1371/journal.pone.0201326 (PMC6188639; doi:10.1371/journal.pone.0201326)
Supplement: S9 File — (PDF) [file pone.0201326.s009.pdf]

Supporting Information file S9:

S9. Toolbox-oriented matrix properties

These properties are useful in the implementation of the model for large images or multiple image patches. These properties are particularly convenient in `Matlab` since it is not very efficient in building large diagonal matrices.

S9.1 Products

Hadamard product and diagonal matrices. Useful to define divisive normalization (e.g. in Eq. 10)

$$\texttt{a.*b} \;=\; \boldsymbol{a} \odot \boldsymbol{b} \;=\; \boldsymbol{b} \odot \boldsymbol{a} \;=\; \mathbb{D}_{\boldsymbol{a}} \cdot \boldsymbol{b} \;=\; \mathbb{D}_{\boldsymbol{b}} \cdot \boldsymbol{a} \quad \text{with} \quad \boldsymbol{a}, \boldsymbol{b} \in \mathbb{R}^{d\times 1}, \quad \mathbb{D}_{\boldsymbol{a}}, \mathbb{D}_{\boldsymbol{b}} \in \mathbb{R}^{d\times d} \tag{S9.1}$$

Kronecker product and matrix replication. This non-commutative product is useful to avoid the large diagonal matrices below.

$$\texttt{repmat(A,m,n)} \;=\; \texttt{kron(ones(m,n),A)} \;=\; \mathbb{1}_{m\times n} \otimes A \tag{S9.2}$$

S9.2 Large matrices (single image patch)

Left-multiplication by diagonal: weight each *row* of A by the corresponding v_i (e.g. in Result I, Eq. 24):

$$\mathbb{D}_{\boldsymbol{v}} \cdot A \;=\; (\mathbb{1}_{1\times d} \otimes \boldsymbol{v}) \odot A \;=\; \texttt{repmat(v,1,d).*A} \tag{S9.3}$$

Right-multiplication by diagonal: weight each *column* of A by the corresponding v_i:

$$A \cdot \mathbb{D}_{\boldsymbol{v}} \;=\; \left(\mathbb{1}_{d\times 1} \otimes \boldsymbol{v}^\top\right) \odot A \;=\; \texttt{repmat(v',d,1).*A} \tag{S9.4}$$

Large block-diagonal matrices in $\nabla_H S$: When dealing with derivatives w.r.t. non-parametric kernels in Result II (either to optimize the kernel or to compute the effect of perturbations) one find products involving huge block-diagonal matrices. These can be avoided:

- In optimization (use Eq. 32 in 48), `deltaS_times_blk_diagJ.m`:

$$\boldsymbol{a}^\top \cdot \mathbb{B}^d_{\boldsymbol{v}^\top} = (\mathbb{1}_{1\times d_v} \otimes \boldsymbol{a}) \odot \left(\mathbb{1}_{d\times 1} \otimes \boldsymbol{v}^\top\right) \tag{S9.5}$$

- In perturbations due to ΔH (apply 32), `blk_diagJ_times_deltaH.m`:

$$\mathbb{B}^d_{\boldsymbol{v}^\top} \cdot \text{vect}(\Delta H^\top) = diag\left(\left(\mathbb{1}_{d\times 1} \otimes \boldsymbol{v}^\top\right) \cdot \Delta H^\top\right) \tag{S9.6}$$

S9.3 Even larger matrices (multiple image patches)

This is how different expressions change when working with N image vectors at the same time (stacked in a single matrix of size $d \times N$) as done by `im2col.m`.

Single vector	$\xrightarrow{\text{stack N vectors}}$	Multiple vectors
$\boldsymbol{v} \in \mathbb{R}^{d\times 1}$	$\longrightarrow$	$\boldsymbol{v} = \begin{pmatrix} \vdots & \vdots & & \vdots \\ \boldsymbol{v}^{[1]} & \boldsymbol{v}^{[2]} & \cdots & \boldsymbol{v}^{[N]} \\ \vdots & \vdots & & \vdots \end{pmatrix} \in \mathbb{R}^{d\times N}$ and $\text{vect}(\boldsymbol{v}) = \begin{pmatrix} \boldsymbol{v}^{[1]} \\ \boldsymbol{v}^{[2]} \\ \vdots \\ \boldsymbol{v}^{[N]} \end{pmatrix} \in \mathbb{R}^{(Nd)\times 1}$
$\Delta\boldsymbol{x}^i = \nabla_{\boldsymbol{x}^{i-1}} S^{(i)} \cdot \Delta\boldsymbol{x}^{i-1}$ where $\nabla_{\boldsymbol{x}^{i-1}} S^{(i)} \in \mathbb{R}^{d_i \times d_{i-1}}$	$\longrightarrow$	$\text{vect}\left(\Delta\boldsymbol{x}^i\right) = \nabla_{\boldsymbol{x}^{i-1}} S^{(i)} \cdot \text{vect}\left(\Delta\boldsymbol{x}^{i-1}\right)$ where $\nabla_{\boldsymbol{x}^{i-1}} S^{(i)} \in \mathbb{R}^{Nd_i \times Nd_{i-1}}$ is block-diag. but stored by convenience as $\in \mathbb{R}^{(Nd_i)\times d_{i-1}}$
$\Delta\boldsymbol{x}^i = \nabla_{\boldsymbol{\Theta}^i} S^{(i)} \cdot \Delta\boldsymbol{\Theta}^i$ where $\nabla_{\boldsymbol{\Theta}^i} S^{(i)} \in \mathbb{R}^{d_i \times d_{\Theta^i}}$	$\longrightarrow$	$\text{vect}\left(\Delta\boldsymbol{x}^i\right) = \nabla_{\boldsymbol{\Theta}^i} S^{(i)} \cdot \Delta\boldsymbol{\Theta}^i$ where $\nabla_{\boldsymbol{\Theta}^i} S^{(i)} \in \mathbb{R}^{(Nd_i)\times d_{\Theta^i}}$
$\mathbb{D}_{\boldsymbol{v}} \cdot A$	$\longrightarrow$	$(\mathbb{1}_{1\times d} \otimes \text{vect}(\boldsymbol{v})) \odot (\mathbb{1}_{N\times 1} \otimes A)$
$A \cdot \mathbb{D}_{\boldsymbol{v}}$	$\longrightarrow$	$\left(\boldsymbol{v}^\top \otimes \mathbb{1}_{d\times 1}\right) \odot (\mathbb{1}_{N\times 1} \otimes A)$
